# Cross-dimensional cultural identity: a multi-dimensional empirical investigation of VTuber audience behavior through the lens of theory of planned behavior

**DOI:** 10.3389/fpsyg.2026.1675447

**Published:** 2026-05-13

**Authors:** Yuan-Ming Chang, Juan Wu, Yan-Xin Hu, Shih-Chih Chen

**Affiliations:** 1 Hankuk University of Foreign Studies, Dongdaemun-gu, Republic of Korea; 2 International Education College, Shandong Jiaotong University, Jinan, China; 3 National Kaohsiung University of Science and Technology, Kaohsiung, Taiwan

**Keywords:** ACG culture identity, content professionalism, sense of virtual community, theory of planned behavior, viewing intention, virtual YouTuber, virtual YouTuber audiences

## Abstract

**Introduction:**

This study employs the Theory of Planned Behavior (TPB) to investigate the key factors influencing audience intention to watch Virtual YouTubers (VTubers). Guided by the decomposition logic of the decomposed theory of planned behavior, twelve antecedent variables were systematically integrated to decompose the three core TPB dimensions—attitude, subjective norms, and perceived behavioral control—within VTuber consumption contexts. Among these, three domain-specific variables—virtual identity identification, ACG culture identity, and cross-cultural influence—were introduced to capture the unique cultural and psychological characteristics of VTuber audiences.

**Methods:**

Data were collected through an online survey platform, yielding 352 valid responses, and analyzed using Structural Equation Modeling (SEM).

**Results:**

The findings reveal that audience attitude and subjective norms significantly and positively influence watching intention, while perceived behavioral control exhibits a relatively weaker effect. Content professionalism and sense of virtual community emerge as the primary antecedents affecting attitude and subjective norms, indicating that audiences prioritize content quality and community connections over virtual identity identification. Time availability proves to be the most crucial determinant of perceived behavioral control, reflecting the constraints of attention economy on VTuber viewing behavior.

**Discussion:**

This study represents the first systematic application of DTPB in quantitative empirical research within the VTuber domain. Beyond contextual application, the findings reveal important boundary conditions: the unexpectedly weak effect of virtual identity identification challenges prevailing assumptions about avatar-based consumption, while the dominance of time constraints over economic barriers provides empirical evidence for attention economy dynamics in virtual entertainment contexts. The results offer both theoretical insights into TPB’s applicability boundaries in emerging virtual content domains and practical implications for platform operation and content design strategies.

## 1 Introduction

Virtual YouTubers (VTubers)—animated digital personas operated by human performers in real-time—have emerged as a compelling phenomenon at the intersection of technology, entertainment, and culture. By combining human authenticity with virtual representation, VTubers offer a unique lens through which to examine how audiences form connections with hybrid human-virtual entities, making them a particularly fascinating subject for scholarly analysis. The digital entertainment landscape has witnessed unprecedented transformation through the proliferation of social media influencers, who leverage online platforms to build audiences and shape consumer behaviors (Belanche et al., 2020). Within this broader ecosystem, livestreaming has emerged as a particularly dynamic medium, fostering real-time interaction between content creators and audiences that transcends traditional unidirectional media consumption models (Hilvert-Bruce et al., 2018). Streamers, defined as content creators who broadcast live video content to audiences in real-time, differ fundamentally from social media influencers through their emphasis on synchronous interaction, community building, and sustained engagement rather than polished, asynchronous content distribution (Sjöblom and Hamari, 2017). This distinction creates unique audience dynamics characterized by heightened parasocial relationships, collective participation, and temporal co-presence that significantly influence viewer behavior and loyalty (Hu et al., 2017).

Within the streaming domain, Virtual YouTubers (VTubers) represent a distinctive subcategory of virtual influencers who utilize animated avatars controlled by human performers to create content and interact with audiences. Unlike traditional virtual influencers that are typically computer-generated entities with predetermined personalities, VTubers combine human agency with virtual representation, creating hybrid digital personas that enable authentic real-time interaction while maintaining fictional character consistency (Stein et al., 2024). The phenomenon has particularly flourished in East Asian markets, where cultural affinity for anime, comics, and games (ACG) provides fertile ground for VTuber adoption and community formation (Tang et al., 2025).

Given the centrality of platform ecosystems in shaping VTuber culture and audience engagement, understanding platform-specific characteristics becomes essential for examining viewing behavior. Bilibili, the focal platform of this study, represents China’s leading video-sharing and livestreaming platform with over 330 million monthly active users as of 2024. Distinguished from global platforms like YouTube or Twitch, Bilibili features unique affordances including Danmaku (real-time bullet screen comments), extensive ACG content curation, and sophisticated community features that foster deep cultural engagement among younger Chinese audiences (Lu et al., 2021). The platform’s recommendation algorithms prioritize community interaction and cultural resonance over pure engagement metrics, creating distinct viewing patterns that emphasize collective participation and cultural identity formation. These platform-specific characteristics make Bilibili particularly relevant for VTuber research, as its technical infrastructure and cultural environment closely align with the interactive, community-driven nature of VTuber content consumption.

Despite extensive research on social media influencers and virtual influencers, significant theoretical gaps persist in understanding VTuber audience behavior mechanisms. While the Theory of Planned Behavior (TPB) has been extensively applied to examine behavioral intentions across various digital contexts (Ajzen, 1991, 2020), its application to VTuber viewing behavior remains theoretically underexplored. Existing TPB applications in influencer research typically focus on isolated platform contexts without adequately addressing the unique psychological and cultural dynamics inherent in VTuber consumption (Park and Smith, 2017).

Three critical gaps emerge from the current literature. First, while studies have examined individual antecedents of attitude, subjective norms, and perceived behavioral control in influencer contexts (Chen and Lin, 2018; Hilvert-Bruce et al., 2018), no comprehensive framework integrates the specific cultural, technological, and social factors that uniquely characterize VTuber audience behavior. The hybrid nature of VTubers—combining human agency with virtual representation—creates novel identification processes that differ substantially from both human influencers and purely virtual entities, necessitating distinct theoretical treatment (Jin, 2012; Wan and Lu, 2024). Second, existing research has not adequately addressed the cross-cultural influence mechanisms that distinguish VTuber consumption from traditional influencer engagement. VTubers frequently transcend national and cultural boundaries through their anime-aesthetic appeal, creating viewing communities that span diverse cultural backgrounds and potentially altering traditional models of social influence and norm formation (Tang et al., 2025). This cross-cultural dimension introduces complexity to subjective norm formation that remains theoretically unaddressed in current TPB applications. Third, the specific technological and resource constraints that govern VTuber viewing behavior have not been systematically integrated into theoretical frameworks for examining viewing intentions. Unlike traditional social media consumption, VTuber engagement often requires sustained time investment, specialized platform navigation skills, and economic resources for community participation, creating unique perceived behavioral control dynamics that warrant distinct theoretical consideration (Lu et al., 2018; Yu et al., 2018).

This study aims to address these gaps by examining how VTuber-specific cultural, technological, and social factors influence audiences’ viewing intentions through the lens of the Theory of Planned Behavior (TPB). Building upon the foundational TPB framework, which posits that behavioral intentions are shaped by attitude, subjective norms, and perceived behavioral control (Ajzen, 1991), and guided by the decomposition logic of the decomposed theory of planned behavior (DTPB; Taylor and Todd, 1995), this research contextualizes TPB within VTuber consumption by systematically decomposing its three core dimensions through twelve domain-specific antecedent variables. While several of these antecedents—such as perceived entertainment, interaction quality, and peer influence—have been examined in broader livestreaming and influencer research, their integration within a unified TPB framework for VTuber contexts is novel. More importantly, three variables—virtual identity identification, ACG culture identity, and cross-cultural influence—are introduced as conceptually distinct constructs that capture the unique hybrid-identity, subcultural, and transnational characteristics of VTuber consumption, serving as boundary condition tests for TPB’s applicability in this emerging domain. Guided by the theoretical logic underlying each TPB dimension, these antecedents are organized into three categories: (1) content- and interaction-related factors—Perceived Entertainment, Virtual Identity Identification, Interaction Quality, and Content Professionalism—that shape audience attitude through the evaluative appraisal of content characteristics and audience-VTuber interaction dynamics; (2) community- and context-related factors—Peer Influence, Sense of Virtual Community, ACG Cultural Identity, and Cross-cultural Influence—that influence subjective norms through the community-based and culturally-embedded nature of VTuber fandom, extending beyond conventional reference groups to encompass virtual communities and subcultural affiliations; and (3) platform- and resource-related factors—Viewing Convenience, Economic Capability, Time Availability, and Platform Accessibility—that determine perceived behavioral control through practical enablers and constraints associated with VTuber viewing on platforms such as Bilibili. This classification follows the theoretical rationale that each category of antecedents corresponds to a distinct psychological mechanism within TPB: attitude formation through behavioral belief evaluation, normative pressure through social and cultural reference groups, and control perception through resource and capability assessment. The detailed theoretical justification for each antecedent variable and its corresponding hypothesis is presented in Sections 2.2 through 2.4. Table 1 illustrates the theoretical gaps in existing TPB applications to livestreaming and virtual influencer research.

**Table 1:** Literature on the use of TPB in the study of live streaming audience behavior and virtual idol fans.

Authors	Analytical method	Research subject	Key findings
Sjöblom et al. (2017)	Questionnaire survey, structural equation modeling	Twitch viewers	Key findings indicate that cognition, affect, interpersonal fusion, social integration, and stress relief are the five primary motivations influencing viewer behavior. Social integration significantly correlates with subscription behavior, while stress relief is strongly associated with viewing duration.
Lu et al. (2018)	Mixed methods (questionnaire + interviews)	Livestreaming users	Chinese livestreaming features diverse content types, with virtual gifting systems and fan group chats serving as crucial social interaction mechanisms. Viewers prefer watching strangers over friends, primarily for entertainment and social needs.
Lee and Watkins (2016)	Structural equation modeling	YouTube users	Video bloggers’ attractiveness and homophily enhance parasocial interaction with viewers, subsequently increasing perceived luxury brand value and purchase intentions, with effects amplifying post-viewing.
Hilvert-Bruce et al. (2018)	Multiple regression analysis	Twitch users	Six primary motivations drive livestream participation: social interaction, community belonging, new friendship formation, entertainment, information acquisition, and lack of real-life support. Compared to mass media, livestream audience participation motivations emphasize social and community foundations.
Hu et al. (2017)	Structural equation modeling	Douyu TV and YY Live users	Dual identification with streamers and viewer communities positively influences continued viewing intention. Streamer identification is influenced by parasocial interaction and self-congruity, while group identification is affected by engagement, cognitive resonance, and collective effervescence. Effects vary across streaming types.
Park and Lin (2020)	Structural equation modeling	Chinese fashion product livestream viewers	Product-source fit influences streamer attractiveness and credibility, while product-content fit affects viewers’ utilitarian and hedonic attitudes. Streamer credibility, hedonic attitudes, and self-product congruence enhance purchase intentions.
Wongkitrungrueng and Assarut (2018)	Partial least squares structural equation modeling	Thai Facebook Live viewers	Livestreaming’s symbolic value directly and indirectly influences consumer engagement, mediated by trust. Utilitarian and hedonic values indirectly affect engagement through product and seller trust.
Yu et al. (2018)	Binary logistic regression and negative binomial regression analysis	AfreecaTV platform users	Viewer engagement positively correlates with gifting behavior. Chat participation and content recommendations positively influence gifting decisions. Interaction with specific streamers significantly affects gift amounts, highlighting fan relationship importance. Gifting behavior varies across content types.
Chen and Lin (2018)	Structural equation modeling	Taiwanese livestream viewers	Entertainment is the primary viewing motivation, followed by streamer appeal. Gender differences are significant: females prioritize streamer appeal, males value interaction. Younger viewers (under 25) are more susceptible to immersion, while older viewers remain unaffected. 65% access livestreams through social media.
Li et al. (2024)	Questionnaire survey, structural equation modeling	VTuber viewers	VTuber’ interactivity and novelty significantly impact user viewing experience and attention, subsequently influencing continued watching intention.

This research makes three primary theoretical contributions to digital consumer behavior and virtual entertainment literature. First, this study provides the first systematic contextual application of DTPB in VTuber audience research. While the decomposition of TPB dimensions into specific antecedent variables follows the established DTPB logic (Taylor and Todd, 1995), the selection and configuration of twelve antecedent variables is specifically tailored to the VTuber consumption context. Among these, nine variables—perceived entertainment, interaction quality, content professionalism, peer influence, sense of virtual community, viewing convenience, economic capability, time availability, and platform accessibility—represent established constructs from livestreaming and digital consumer research that are recontextualized for VTuber audiences, while three variables—virtual identity identification, ACG culture identity, and cross-cultural influence—are conceptually novel constructs that capture the distinctive hybrid-identity, subcultural, and transnational dimensions of VTuber consumption. This differentiated integration provides a comprehensive yet contextually grounded framework for understanding the multi-layered influences on VTuber viewing intentions. Second, the study identifies important boundary conditions for TPB’s applicability in virtual entertainment contexts. The empirical findings reveal that virtual identity identification—a construct theoretically central to avatar-based media consumption—exerts a surprisingly weak influence on audience attitude compared to content professionalism and interaction quality. This counterintuitive finding challenges prevailing assumptions that virtual character identification dominates VTuber engagement, suggesting that audiences approach VTubers primarily as content creators rather than identity extensions. Similarly, the finding that time availability substantially exceeds economic capability as the primary determinant of perceived behavioral control provides empirical evidence for the shift from economic-constrained to attention-constrained consumption in digital entertainment, revealing boundary conditions that differentiate VTuber viewing from traditional media consumption models. Third, the study advances cross-cultural consumer behavior theory by empirically demonstrating how virtual content transcends traditional cultural boundaries to influence viewing behavior. Through the integration of cross-cultural influence and ACG culture identity as predictors of subjective norms, we illuminate novel pathways through which global virtual content shapes local consumption patterns, contributing to theoretical understanding of cultural convergence in digital environments. From a managerial perspective, the findings offer strategic guidance for platform operators, VTuber agencies, and content creators seeking to enhance audience engagement and retention. By identifying the relative importance of content professionalism, community building, and resource accessibility factors, the research provides evidence-based recommendations for optimizing VTuber operational strategies and platform design.

## 2 Theoretical background and hypotheses development

### 2.1 Theory of planned behavior

Since its inception, TPB has become one of the most influential and widely applied theoretical frameworks for understanding and explaining human behavior across diverse domains (Armitage and Conner, 2001). Meta-analytic reviews have consistently demonstrated TPB’s robust predictive validity, with the theory accounting for substantial variance in both behavioral intentions and actual behaviors across health, environmental, consumer, and technology adoption contexts (Armitage and Conner, 2001; Hagger et al., 2002).

The first core construct, attitude toward behavior, represents an individual’s evaluative predisposition toward performing specific behaviors, encompassing both cognitive assessments of behavioral outcomes and affective responses to behavioral engagement (Taufik et al., 2016). This construct captures the degree to which individuals hold favorable or unfavorable evaluations of the target behavior based on their beliefs about its consequences. In livestreaming contexts, research demonstrates that user attitudes are significantly shaped by content quality, streamer authenticity, and interactive experiences, with positive attitudes serving as strong predictors of continued engagement (Park and Lin, 2020; Hilvert-Bruce et al., 2018). Within the VTuber domain, audience attitudes manifest through comprehensive evaluations of virtual character appeal, content entertainment value, and overall viewing satisfaction, reflecting users’ cognitive and emotional responses to the unique blend of virtual representation and human performance that characterizes VTuber content.

The second construct, subjective norms, encompasses the perceived social pressure and expectations from significant reference groups regarding behavioral performance (Park and Smith, 2017). This construct operates through two distinct mechanisms: descriptive norms, which reflect perceptions of others’ actual behaviors, and injunctive norms, which capture perceived expectations and approval from important others. Research in digital entertainment contexts reveals that subjective norms exert particularly strong influences on user participation, as community atmosphere and peer behaviors significantly shape individual engagement patterns (Sjöblom and Hamari, 2017; Lu et al., 2018). The communal nature of VTuber consumption, characterized by shared viewing experiences, fan community participation, and collective cultural identity formation, amplifies the relevance of subjective norms in predicting viewing intentions.

The third construct, perceived behavioral control, reflects individuals’ assessments of their capability to perform the target behavior, encompassing both internal capabilities and external facilitating conditions (Ajzen, 1991). This construct captures the perceived ease or difficulty of behavioral execution, influenced by factors such as resource availability, skill requirements, and environmental constraints (Wan and Chiou, 2016). In digital content consumption contexts, perceived behavioral control encompasses technical competencies, platform familiarity, resource accessibility, and temporal flexibility that collectively determine users’ confidence in successfully engaging with content (Wongkitrungrueng and Assarut, 2018; Chen and Lin, 2018). For VTuber viewing behavior, this construct assumes particular importance given the potential barriers related to platform navigation, time investment requirements, economic resources for community participation, and cultural familiarity with anime-style content.

The dependent variable, behavioral intention, represents an individual’s subjective likelihood of attempting to perform a specific behavior, serving as the most proximal cognitive antecedent of actual behavioral execution (Ajzen, 1991). In VTuber research contexts, watching intention operationalizes this concept by capturing users’ motivational readiness to engage in sustained viewing behaviors, influenced by content quality perceptions, social interaction needs, and flow experiences that characterize immersive digital entertainment consumption (Chen and Lin, 2018; Li et al., 2024). Research demonstrates that watching intentions strongly predict subsequent engagement behaviors, including sustained viewing, community participation, and financial support activities that sustain the VTuber ecosystem (Yu et al., 2018; Hu et al., 2017).

Applied to the VTuber context on Bilibili platform, these constructs provide a comprehensive framework for understanding the psychological mechanisms underlying audience viewing decisions. The integration of TPB’s three core constructs enables systematic examination of how cognitive evaluations (attitude), social influences (subjective norms), and resource assessments (perceived behavioral control) collectively shape users’ intentions to engage with VTuber content, thereby providing theoretical foundation for predicting and understanding audience behavior in this emerging digital entertainment domain. It is important to note the theoretical positioning of the present study in relation to the decomposed theory of planned behavior (DTPB). Taylor and Todd (1995) proposed the DTPB by decomposing attitude, subjective norms, and perceived behavioral control into more specific belief dimensions, thereby enhancing predictive specificity while maintaining TPB’s parsimonious theoretical structure. The present study adopts this decomposition logic as its methodological foundation, systematically identifying domain-specific antecedent variables for each TPB dimension within VTuber consumption contexts. However, this study is not a mechanical application of the original DTPB decomposition categories (e.g., perceived usefulness, compatibility, and ease of use for attitude). Rather, the specific antecedent variables are theoretically derived from the unique characteristics of VTuber consumption—particularly the hybrid human-virtual identity dynamics, subcultural embeddedness in ACG culture, and cross-cultural content flows—that distinguish this domain from the technology adoption contexts in which DTPB was originally developed. In this regard, the present research represents a context-specific DTPB application that simultaneously examines the boundary conditions under which established antecedent relationships may operate differently in VTuber consumption compared to other digital entertainment contexts.

### 2.2 Content- and interaction-related factors

This section examines four antecedent variables that collectively shape audience attitude toward VTuber viewing: Perceived Entertainment, Virtual Identity Identification, Interaction Quality, and Content Professionalism. These variables share a common theoretical logic grounded in the evaluative appraisal of content characteristics and audience-VTuber interaction dynamics. According to TPB, attitude represents an individual’s positive or negative evaluation of performing a specific behavior, formed through behavioral beliefs about the outcomes of that behavior (Ajzen, 1991). In the VTuber consumption context, attitude formation is driven by audiences’ assessments of what VTuber content offers—including hedonic gratification, psychological identification with virtual personas, the quality of real-time interactive exchanges, and the professional standards of content production. Each of these factors represents a distinct facet of the content consumption experience that contributes to the overall attitudinal evaluation. Perceived Entertainment (PE) is operationally defined as the degree to which audiences perceive VTuber content as providing amusement, enjoyment, and relaxation (Vorderer et al., 2004; Sherry, 2004). This construct encompasses the subjective evaluation of content’s capacity to generate positive emotional responses and leisure satisfaction among viewers. As a core characteristic of VTuber livestreaming, PE significantly and positively influences Audience Attitude (AA). Research indicates that entertainment value is a key factor affecting AA and continued viewing intention (Hu et al., 2017), positively predicting user attitudes (Chen and Lin, 2018). This finding has been further supported by cross-platform studies (Lu et al., 2018; Zhao et al., 2018). From a psychological mechanism perspective, entertainment experiences influence AA through emotional engagement (Lim et al., 2020; Wohn et al., 2018; Hilvert-Bruce et al., 2018). Therefore, this study proposes the following hypothesis:

● H1: Perceived entertainment positively impacts audience attitudes.

Virtual Identity Identification (VII) is operationally defined as the degree to which audiences recognize and emotionally connect with VTuber virtual personas, encompassing both cognitive acceptance of the virtual character and affective attachment to their digital identity (Jin, 2012; Tajfel, 1982). This construct captures the psychological process through which viewers develop identificatory relationships with animated avatars, transcending traditional human-to-human parasocial interactions. VII serves as a crucial psychological mechanism in VTuber-audience interactions, influencing the formation of Audience Attitude. Jin (2012) virtual identity discrepancy model explicates the process through which audiences construct social relationships by identifying with virtual personas, a framework validated by Wan and Lu (2024). From a social cognitive perspective, identification influences audience attitudes through emotional engagement (Lim et al., 2020; Cohen and Tyler, 2016). Jin and Martin (2015) demonstrate that virtual identity characteristics significantly impact users’ cognitive evaluations. Therefore, this study proposes the following hypothesis:

● H2: Virtual identity identification positively impacts audience attitudes.

Interaction Quality (IQ) is operationally defined as the frequency, timeliness, and attentiveness of interactions between VTubers and their audiences, reflecting the overall effectiveness and satisfaction derived from bidirectional communication exchanges (Hamilton et al., 2014; Wongkitrungrueng and Assarut, 2018). This construct encompasses both quantitative aspects (response frequency and speed) and qualitative dimensions (personalization and relevance) of streamer-viewer interactions. As a core characteristic of streamer-audience communication on livestreaming platforms, significantly influences audience attitude. Research demonstrates that high-quality interactions enhance audience attitudes and engagement levels (Lu et al., 2018; Hamilton et al., 2014) and influence supportive behaviors (Wohn et al., 2018). Studies from the user motivation perspective confirm that IQ is a critical factor affecting audience attitudes (Hu et al., 2017; Sjöblom and Hamari, 2017), influencing tipping behavior and usage intention (Yu et al., 2018; Chen and Lin, 2018). Therefore, this study proposes the following hypothesis:

● H3: Interaction quality positively impacts audience attitudes.

Content Professionalism (CP) is operationally defined as the level of professional competence, preparation thoroughness, and overall quality demonstrated in VTuber content creation, encompassing technical execution, content depth, and presentation standards (Lee and Watkins, 2016; Park and Lin, 2020). This construct reflects the creator’s expertise in delivering well-structured, informative, and polished content that meets audience expectations for professional entertainment experiences. As a key indicator measuring content creators’ performance level, significantly influences audience attitude. Research indicates that creators’ professional performance affects audience cognitive evaluations (Lee and Watkins, 2016), with proficiency level significantly impacting audience attitudes and supportive behaviors (Zhang et al., 2019; Maruyama et al., 2017). Studies also confirm that CP positively influences continued viewing intention (Zhao et al., 2018) and fan stickiness (Hu et al., 2020). Accordingly, this study proposes the following hypothesis:

● H4: Content professionalism positively impacts audience attitudes.

### 2.3 Community- and context-related factors

This section shows four antecedent variables that influence subjective norms regarding VTuber viewing behavior: Peer Influence, Sense of Virtual Community, ACG Cultural Identity, and Cross-cultural Influence. These constructs reflect the community-based and culturally-embedded nature of VTuber fandom. Subjective norms in TPB refer to the perceived social pressure to perform or not perform a behavior, stemming from normative beliefs about the expectations of important referent groups (Ajzen, 1991). While traditional TPB applications typically focus on interpersonal influence from family, friends, and colleagues, VTuber consumption occurs within distinctive social contexts that extend beyond conventional reference groups. The normative environment of VTuber audiences encompasses not only immediate peer networks but also virtual fan communities, subcultural affiliations rooted in ACG culture, and transnational cultural flows that create normative pressures transcending local social contexts. Research on social identity theory suggests that when individuals identify with a social group, they tend to adopt the values and norms of that group, which subsequently influences their behavioral intentions (Cheung and Lee, 2010; Zhou, 2011). Peer Influence (PI) is operationally defined as the degree of supportive attitudes, social pressure, and behavioral influence exerted by one’s social circle regarding VTuber viewing behaviors, reflecting the extent to which friends and peers shape individual consumption decisions through direct recommendations, approval expressions, and shared viewing practices (Deutsch and Gerard, 1955; Jung et al., 2012). This construct captures both explicit peer recommendations and implicit social pressures that emerge from group dynamics within social networks. In the context of VTuber culture, PI exerts a significant positive effect on Subjective Norm (SN). TPB identifies SN as a key predictor of behavioral intention, primarily influenced by the attitudes and expectations of significant others (Ajzen, 1991). Research demonstrates that individuals tend to conform to peer group values to gain acceptance (Deutsch and Gerard, 1955), with peer behavior significantly influencing users’ subjective norms (Liu et al., 2018) and media content preferences (Jung et al., 2012). Based on this, this study proposes the following hypothesis:

● H5: Peer influence positively impacts subjective norms.

Sense of Virtual Community (SOVC) is operationally defined as the degree of identification and belonging that audiences feel toward VTuber fan communities, encompassing emotional attachment to the group, shared identity with fellow members, and perceived integration within the virtual collective (Blanchard and Markus, 2004; Koh and Kim, 2004). This construct reflects the psychological connection individuals develop with online communities centered around specific VTubers, manifesting through active participation, emotional investment, and alignment with community values and practices. In virtual communities, Sense of Virtual Community (SOVC) positively influences subjective norms. Research indicates that SOVC is a crucial factor in the formation of behavioral norms, with members strengthening their compliance through identification with community norms (Blanchard and Markus, 2004). This sense of belonging facilitates awareness of others’ expectations and reinforces norm internalization through interaction (Koh and Kim, 2004; Dholakia et al., 2004). Member interaction and identification enhance the formation of subjective norms (Shen and Khalifa, 2008; Wang et al., 2012). Based on this, this study proposes the following hypothesis:

● H6: Sense of virtual community positively impacts subjective norms.

ACG Culture Identity (ACGCI) is operationally defined as the degree of participation and intrinsic identification that audiences demonstrate toward anime, comics, and games cultural elements, reflecting their psychological attachment to and behavioral engagement with Japanese-originated popular culture aesthetics, narratives, and community practices (Yee, 2006; Oh et al., 2018). This construct encompasses both active consumption of ACG content and internalization of subcultural values, manifesting through knowledge acquisition, community participation, and lifestyle integration of ACG-related elements. Within virtual communities, cultural identity critically shapes subjective norms, with Social Identity Theory positing that cultural group identification facilitates norm internalization (Tajfel, 1982; Ashforth and Mael, 1989). Research demonstrates that user behavior is closely associated with cultural identity (Yee, 2006), which reinforces behavioral norms through enhanced social perception (Appiah, 2004) and provides a collective sense of belonging in virtual communities (Oh et al., 2018). Within the context of ACG (Anime, Comics, and Games) culture, the VTuber phenomenon provides users with a strong sense of cultural identity, facilitating the formation of subjective norms through group belonging needs and emotional connections. Based on this, this study proposes the following hypothesis:

● H7: ACG culture identity positively impacts subjective norms.

Cross-Cultural Influence (CCI) is operationally defined as the degree of change in individuals’ cultural understanding and communicative behaviors resulting from exposure to different national or regional cultures through VTuber content, encompassing cognitive adaptation to foreign cultural elements, behavioral modification in cross-cultural interactions, and attitudinal shifts toward cultural diversity (Berry, 1997; Markus and Kitayama, 1991). This construct captures the transformative process whereby individuals develop enhanced cultural competence, adopt new communicative practices, and internalize diverse cultural perspectives through sustained engagement with VTuber content that bridges multiple cultural contexts. In the context of globalization and digitalization, perceived cross-cultural exposure through VTuber content serves as a mechanism in shaping subjective norms among audiences within a given cultural context. Research indicates that the transmission of values across cultures can forge new behavioral norms (Hofstede, 1984), establishing shared norms through social networks (Lin and Lu, 2011). Cultural Adaptation Theory and Cultural Convergence Theory emphasize that cross-cultural contact leads individuals to internalize new group norms (Berry, 1997), influencing the formation of subjective norms through enhanced collective consciousness (Markus and Kitayama, 1991). Based on this, this study proposes the following hypothesis:

● H8: Cross-cultural influence positively impacts subjective norms.

### 2.4 Platform- and resource-related factors

This section describes four antecedent variables that determine perceived behavioral control over VTuber viewing: Viewing Convenience, Economic Capability, Time Availability, and Platform Accessibility. Perceived behavioral control in TPB reflects the perceived ease or difficulty of performing a behavior, encompassing both self-efficacy beliefs and perceptions of external facilitating conditions (Ajzen, 2002). The decomposed theory of planned behavior (DTPB) further distinguishes between self-efficacy, resource facilitating conditions, and technology facilitating conditions as distinct components of perceived behavioral control (Taylor and Todd, 1995). Viewing Convenience (VC) is operationally defined as the degree of ease and accessibility that audiences experience in watching VTuber content across physical environments, device conditions, and temporal arrangements, encompassing the flexibility of viewing locations, compatibility across multiple devices, and adaptability to personal schedules (Davis, 1989; Venkatesh et al., 2003). This construct reflects the technological and situational facilitators that enable seamless content consumption, including platform accessibility, mobile compatibility, and temporal flexibility that accommodates diverse lifestyle patterns. In the context of digital content consumption, VC as an external environmental factor plays a significant role in enhancing users’ Perceived Behavioral Control (PBC). TPB suggests that PBC reflects individuals’ perception of resource availability (Ajzen, 1991). Research demonstrates that ease of use influences users’ sense of control (Davis, 1989), with viewing convenience showing a positive association with behavioral control (Venkatesh et al., 2003) and reducing barriers to behavioral execution (Sun and Zhang, 2006). Based on this, this study proposes the following hypothesis:

● H9: Viewing convenience positively impacts perceived behavioral control.

Economic Capability (EC) is operationally defined as the sustainable financial capacity that audiences possess to make economic investments in VTuber-related activities, encompassing their ability to afford basic viewing costs, provide regular financial support, and engage in additional consumption behaviors such as merchandise purchases and premium content access (Taylor and Todd, 1995; Venkatesh et al., 2012). This construct reflects individuals’ discretionary income allocation toward VTuber consumption, their financial resilience to maintain ongoing support commitments, and their capacity to participate in monetized community activities without experiencing economic strain. EC, as a key external resource, has a significant positive influence on individuals’ PBC. TPB indicates that resource availability determines PBC (Ajzen, 1991). Research demonstrates that EC reduces behavioral resistance (Taylor and Todd, 1995), serves as a crucial prerequisite for perceived behavioral feasibility (Venkatesh et al., 2012), and directly affects payment willingness and sense of behavioral control (Homburg et al., 2005). Based on this, this study proposes the following hypothesis:

● H10: Economic capability positively impacts perceived behavioral control.

Time Availability (TA) is operationally defined as the discretionary time resources that audiences can autonomously allocate, regularly invest, and flexibly arrange for VTuber viewing activities within their daily routines, encompassing both scheduled viewing periods and spontaneous engagement opportunities that accommodate personal lifestyle patterns and competing demands (Bandura, 1982; Taylor and Todd, 1995). This construct reflects individuals’ temporal autonomy to pursue sustained engagement with VTuber content, their capacity to maintain consistent viewing schedules, and their flexibility to adapt viewing behaviors to dynamic life circumstances without experiencing time-related stress or conflicts with essential obligations. TA has a significant positive effect on PBC. TPB indicates that TA, as a crucial external condition, enhances the sense of behavioral control (Ajzen, 1991). Research demonstrates that time sufficiency improves perceived behavioral feasibility (Bandura, 1982), reduces behavioral execution barriers (Taylor and Todd, 1995), and serves as a core element in strengthening perceived control (Venkatesh et al., 2012). This is particularly important in activities requiring sustained engagement (Cheung and Lee, 2010). Based on this, this study proposes the following hypothesis:

● H11: Time availability positively impacts perceived behavioral control.

Platform Accessibility (PA) is operationally defined as the functional completeness and user-friendliness that Bilibili platform provides at the technical level for delivering VTuber content services to users, encompassing interface intuitiveness, navigation efficiency, search functionality effectiveness, content discovery mechanisms, and cross-device compatibility that facilitate seamless user interactions (Davis, 1989; Venkatesh et al., 2003). This construct reflects the platform’s technical infrastructure quality, including loading speed, system stability, feature availability, and adaptive design that collectively reduce cognitive load and operational complexity for users accessing VTuber content. PA, as a vital external resource, exerts a significant positive influence on individuals’ PBC. TPB identifies PA as a core determinant of behavioral control (Ajzen, 1991). Research demonstrates that system usability reduces usage barriers (Davis, 1989), with PA serving as a crucial factor in enhancing perceived control (Venkatesh et al., 2003), while user-friendly interfaces minimize operational burden (Gefen and Straub, 2000). Based on this, this study proposes the following hypothesis:

● H12: Platform accessibility positively impacts perceived behavioral control.

### 2.5 Logical factors of watching intention

Audience Attitude is operationally defined as the positive or negative evaluative judgments that Bilibili users hold toward watching VTuber livestreams and videos, encompassing affective responses, cognitive assessments, and overall favorability toward VTuber content consumption experiences (Fishbein and Ajzen, 1977; Davis, 1989). Subjective Norm refers to users’ perceptions of social pressure from significant others regarding their VTuber viewing behaviors, including perceived expectations, approval, and recommendations from family members, friends, and online community members (Kim et al., 2011; Venkatesh et al., 2003). Perceived Behavioral Control represents users’ sense of control over their ability to successfully watch and participate in VTuber-related activities, reflecting their confidence in overcoming potential barriers and executing desired viewing behaviors (Taylor and Todd, 1995; Pavlou and Fygenson, 2006). Watch Intention is defined as the subjective willingness and commitment that Bilibili users demonstrate toward continuously watching and following VTuber content, indicating their motivational readiness to engage in sustained viewing behaviors (Lee and Watkins, 2016).

TPB identifies attitude, subjective norms, and perceived behavioral control as core variables in predicting behavioral intentions (Ajzen, 1991). Attitude reflects individual evaluations of behavior, with positive attitudes significantly enhancing behavioral intentions (Fishbein and Ajzen, 1977; Davis, 1989). In virtual experience contexts, viewers’ positive attitudes toward virtual influencers enhance their viewing intentions (Luo et al., 2011; Hollebeek and Macky, 2019). Subjective norms represent perceived social pressure, with particularly strong effects in collectivist cultures (Kim et al., 2011; Venkatesh et al., 2003). In digital content consumption, fan community recommendations and social interactions strengthen viewing intentions (Lee and Watkins, 2016). Perceived behavioral control reflects the perceived ease of behavioral execution, with higher control enhancing users’ behavioral confidence (Taylor and Todd, 1995). In VTuber viewing contexts, technological usability and platform convenience, as sources of control perception, significantly increase viewing intentions (Pavlou and Fygenson, 2006). Collectively, these three variables operate through distinct pathways to positively influence VTuber audience viewing intentions. Based on this, the following hypothesis is proposed:

● H13: Audience attitude positively impacts watching intention.

● H14: Subjective norm positively impacts watching intention.

● H15: Perceived behavioral control positively impacts watching intention.

**Figure 1:**
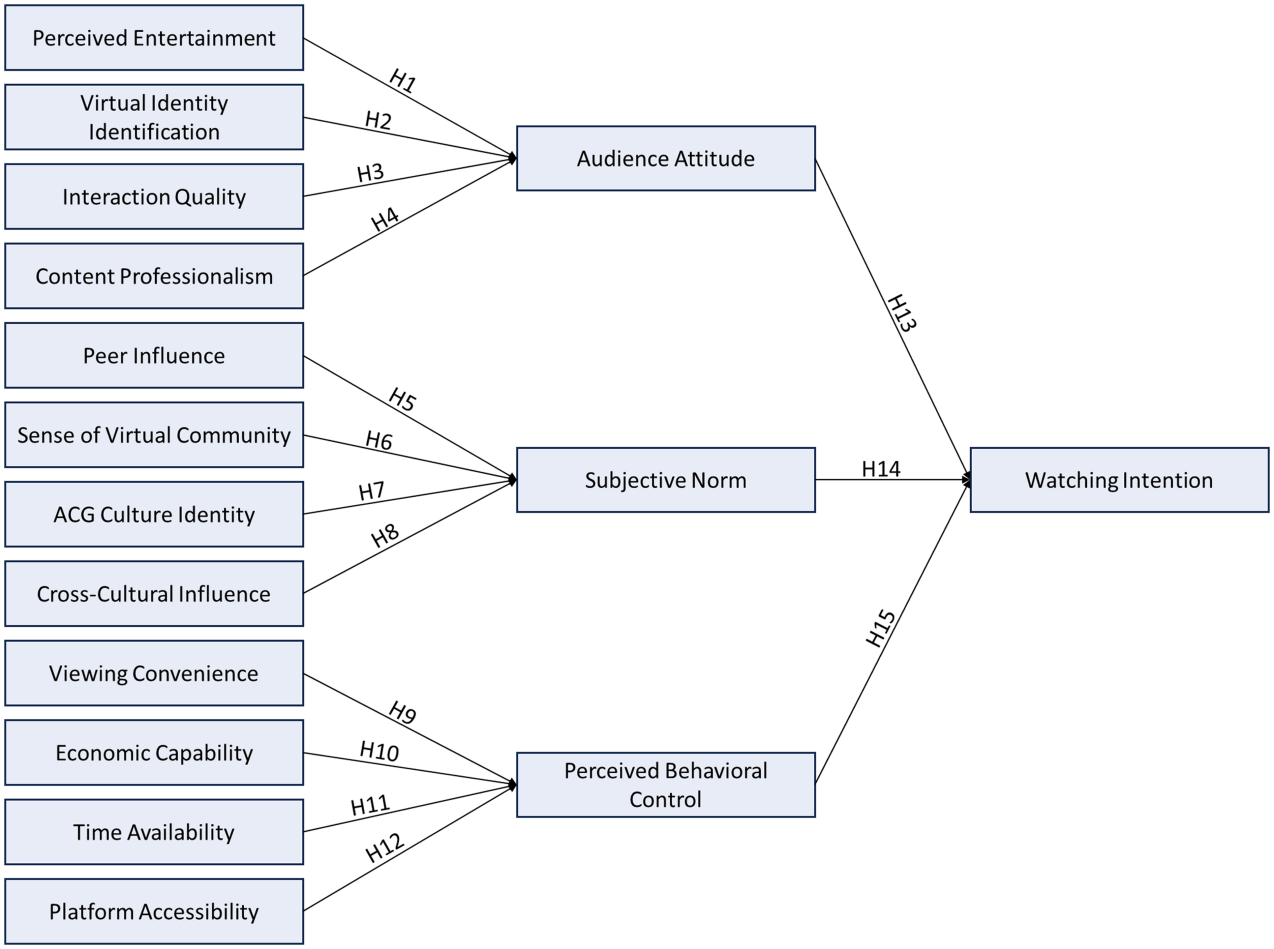
Research model.

The research model based on these hypotheses is illustrated in Figure 1.

## 3 Research methodology and sample structure

Data collection for this study was conducted through a questionnaire survey, with research indicators, theoretical framework, and operational definitions derived from established hypotheses and supported by relevant literature. While most measurement items were adapted from existing research to ensure content validity, we conducted pilot testing with university students and refined the measurement items based on their feedback. To ensure instrument validity and reliability, a multi-stage instrument development process was followed. First, regarding content validity, all measurement items were initially drawn from well-established scales in prior livestreaming, virtual influencer, and consumer behavior research. Specifically, perceived entertainment items were adapted from Hu et al. (2017) and Vorderer et al. (2004); virtual identity identification from Jin (2012) and Tajfel (1982); interaction quality from Hamilton et al. (2014) and Wongkitrungrueng and Assarut (2018); content professionalism from Lee and Watkins (2016) and Park and Lin (2020); peer influence from Deutsch and Gerard (1955) and Jung et al. (2012); sense of virtual community from Blanchard and Markus (2004) and Koh and Kim (2004); ACG cultural identity from Yee (2006) and Oh et al. (2018); cross-cultural influence from Berry (1997) and Markus and Kitayama (1991); viewing convenience, economic capability, time availability, and platform accessibility from Davis (1989), Taylor and Todd (1995), and Venkatesh et al. (2003, 2012); and the core TPB constructs of attitude, subjective norms, perceived behavioral control, and watching intention from Ajzen (1991), Fishbein and Ajzen (1977), and Kim et al. (2011). Items were translated into Chinese using a back-translation procedure (Brislin, 1970) to ensure linguistic equivalence. Second, a panel of three scholars with expertise in digital media, consumer behavior, and survey methodology reviewed the draft instrument for item clarity, conceptual relevance, and cross-cultural appropriateness, resulting in the revision of five items that were deemed ambiguous or insufficiently aligned with the VTuber consumption context. Third, a pilot study was conducted with 45 university students who were active VTuber viewers on Bilibili. The pilot data were examined for item-total correlations (all exceeding 0.50) and preliminary reliability (Cronbach’s alpha values for all constructs exceeding 0.80), and participant feedback led to minor wording adjustments to improve clarity and cultural relevance. This systematic approach to instrument development enhances confidence in the content validity and initial reliability of the measurement scales employed in the main study.

This study administered an electronic questionnaire through the WJX platform (www.wjx.cn), a leading online survey platform in China with extensive user base and comprehensive data collection capabilities. The decision to employ an online survey methodology was based on three primary considerations: First, this method closely aligns with the research topic, particularly suitable for investigating user behavior and attitudes in online environments; Second, web-based questionnaires enable efficient access to target populations, significantly enhancing sample collection effectiveness; Third, online surveys offer notable advantages in terms of cost and time investment compared to traditional paper-based questionnaires (Sogari et al., 2015). The survey was conducted between March and April 2025, with the research team distributing survey invitation links through multiple WeChat groups across mainland China. To ensure sample representativeness, gender distribution was actively monitored during the sampling process. The final valid sample comprised 56% male and 44% female respondents, effectively reflecting the structural characteristics of the target population (Valentine et al., 2019). A total of 379 questionnaires were collected during the survey period. Rigorous data cleaning procedures were implemented following established methodological guidelines to ensure data quality and reliability (Hair et al., 2022; Ringle et al., 2024). Completeness checks removed questionnaires with missing responses exceeding 10% of total items, following Tabachnick and Fidell (2013) recommendations for acceptable missing data thresholds. Validity checks eliminated responses with completion times below 300 s (representing less than 5 s per item on average) or exceeding 3,600 s, indicating insufficient attention or survey interruption (Meade and Craig, 2012). Straight-line response patterns were identified and removed using variance-based detection methods, eliminating responses with standard deviations below 0.5 across Likert-scale items (DeSimone et al., 2015). Logical consistency checks excluded questionnaires exhibiting contradictory responses to related items, such as claiming no VTuber viewing experience while providing detailed engagement preferences. This process yielded 352 valid questionnaires, representing a response rate of 93%. It should be noted that the recruitment of respondents through WeChat groups constitutes a convenience sampling approach, which may introduce self-selection bias, as participants who voluntarily joined VTuber-related discussion groups are likely to represent more engaged and enthusiastic audience segments (Wright, 2005). This sampling strategy is nonetheless consistent with established practices in online community research, where convenience and snowball sampling through platform-specific social networks remain the predominant approach for accessing hard-to-reach digital subcultures (Baltar and Brunet, 2012). Furthermore, given that Bilibili’s VTuber community is itself organized around interest-based social networks, the use of WeChat groups as recruitment channels closely mirrors the actual social infrastructure through which VTuber audiences communicate and share content, thereby enhancing the ecological validity of the sample for this specific research context. All participants were Chinese nationals residing in mainland China, ensuring cultural and linguistic homogeneity essential for examining ACG culture identity and cross-cultural influence constructs within the Chinese context. Table 2 presents the demographic characteristics of the participants. Among respondents, the 18–35 age group constituted the largest proportion (77.6%), with university graduates representing the highest educational attainment (44.6%), followed by vocational college graduates (26.1%). In terms of occupational distribution, office workers accounted for 27.3%, followed by ACG culture industry professionals (23.6%) and new media/internet practitioners (23.3%). Overall, the sample data effectively reflects China’s social structure while ensuring participants possessed basic knowledge of VTubers.

**Table 2:** Sample demographics.

Characteristics	Description	Frequency	Percent (%)
Gender	Female	155	44.0%
	Male	197	56.0%
Age	Under 17 years old	10	2.8%
	18–25	91	25.6%
	26–30	118	33.5%
	31–35	65	18.5%
	36–40	28	8.0%
	41–45	18	5.1%
	46–50	11	3.1%
	51 years old or above	11	3.1%
Education level	Middle school or lower degree	6	1.7%
	High school certificate (secondary vocational school diploma)	54	15.3%
	Higher vocational schools diploma (junior college)	92	26.1%
	Undergraduate degree	157	44.6%
	Master or higher degree	43	12.2%
Career	Student	10	2.8%
	Office worker	96	27.3%
	Freelancer	21	6.0%
	Anime culture practitioners (workers and creators in industries such as animation, comics, games, etc.)	83	23.6%
	New media and Internet practitioners (such as internet hosts, social media operators, video producers, etc.)	82	23.3%
	VTuber related professions (painters, modelers, etc.)	60	17.1%

Sample size adequacy was evaluated using multiple criteria appropriate for CB-SEM analysis. First, the final sample of 352 respondents substantially exceeds the widely recommended minimum threshold of 200 cases for SEM studies (Kline, 2023). Second, following the a-priori sample size determination approach for SEM developed by Westland (2010), and implemented through Soper (2024) online calculator, with 16 latent variables, 64 observed variables, a medium anticipated effect size of 0.30, a probability level of 0.05, and a statistical power level of 0.80, the minimum recommended sample size was determined to be 215 observations. The current sample of 352 exceeds this requirement by 63.7%, providing sufficient statistical power to detect meaningful effects within the proposed structural model. Third, following the subject-to-variable ratio criterion, the present sample yields a ratio of approximately 5.5:1 (352 cases to 64 observed variables), which meets the minimum threshold of 5:1 widely recommended for confirmatory factor analysis and structural equation modeling (Hair et al., 2019; Bentler and Chou, 1987). Fourth, Wolf et al. (2013) conducted a comprehensive Monte Carlo simulation study demonstrating that for SEM models with standardized factor loadings of 0.65 or higher—a condition satisfied by all indicators in this study (see Table 3)—sample sizes between 150 and 350 are generally sufficient to achieve adequate statistical power (≥ 0.80) for models of moderate complexity. The present sample of 352 therefore satisfies this simulation-based benchmark. Although the sample size is adequate by these multiple criteria, it should be acknowledged that larger samples would further enhance the stability of parameter estimates and increase the precision of model fit indices, particularly for complex models with many parameters (Kline, 2023). This limitation is explicitly addressed in Section 5.3. Data analysis was conducted using IBM SPSS 28 for preliminary descriptive statistics and IBM SPSS AMOS 28 for SEM analysis. The analytical procedure followed the two-stage approach recommended by Anderson and Gerbing (1988), beginning with measurement model assessment followed by structural model evaluation and hypothesis testing.

**Table 3:** Reliability analysis and convergent validity.

Dimensions	Items	Factor loading	*z*-value	AVE	Cronbach’s alpha	Composite reliability
Perceived entertainment	PE1	0.935	143.794	0.875	0.965	0.966
	PE2	0.928	126.515			
	PE3	0.945	143.877			
	PE4	0.933	134.047			
Virtual identity identification	VII1	0.931	122.700	0.869	0.964	0.963
	VII2	0.923	120.335			
	VII3	0.935	125.118			
	VII4	0.939	130.270			
Interaction quality	IQ1	0.931	135.847	0.873	0.965	0.965
	IQ2	0.936	131.535			
	IQ3	0.932	125.115			
	IQ4	0.938	133.227			
Content professionalism	CP1	0.928	124.426	0.867	0.963	0.963
	CP2	0.931	126.487			
	CP3	0.931	133.960			
	CP4	0.934	139.716			
Audience attitude	AA1	0.948	149.722	0.881	0.967	0.967
	AA2	0.940	138.680			
	AA3	0.937	134.287			
	AA4	0.929	135.434			
Peer influence	PI1	0.940	152.957	0.871	0.964	0.964
	PI2	0.931	121.558			
	PI3	0.924	122.415			
	PI4	0.940	133.457			
Sense of virtual community	SOVC1	0.937	130.924	0.875	0.966	0.965
	SOVC2	0.935	140.580			
	SOVC3	0.939	140.028			
	SOVC4	0.931	129.149			
ACG culture identity	ACGCI1	0.931	118.403	0.875	0.966	0.966
	ACGCI2	0.944	141.866			
	ACGCI3	0.929	140.661			
	ACGCI4	0.938	133.842			
Cross-cultural influence	CCI1	0.936	142.224	0.870	0.964	0.964
	CCI2	0.930	120.574			
	CCI3	0.929	123.588			
	CCI4	0.934	138.148			
Subjective norm	SN1	0.933	130.230	0.874	0.965	0.965
	SN2	0.937	139.980			
	SN3	0.937	125.454			
	SN4	0.933	132.320			
Viewing convenience	VC1	0.935	146.803	0.870	0.964	0.964
	VC2	0.937	136.258			
	VC3	0.931	129.049			
	VC4	0.927	122.783			
Economic capability	EC1	0.943	146.046	0.880	0.967	0.967
	EC2	0.935	136.438			
	EC3	0.929	117.562			
	EC4	0.946	172.163			
Time availability	TA1	0.935	128.948	0.871	0.964	0.964
	TA2	0.929	123.249			
	TA3	0.938	139.125			
	TA4	0.930	128.712			
Platform accessibility	PA1	0.939	130.905	0.867	0.963	0.963
	PA2	0.917	107.887			
	PA3	0.937	139.743			
	PA4	0.932	112.515			
Perceived behavioral control	PBC1	0.933	134.608	0.865	0.963	0.963
	PBC2	0.932	118.915			
	PBC3	0.930	130.644			
	PBC4	0.926	110.376			
Watching intention	WI1	0.921	123.793	0.861	0.963	0.961
	WI2	0.932	126.476			
	WI3	0.920	111.635			
	WI4	0.938	153.031			

## 4 Data analysis results

### 4.1 Measurement model

As all data were collected via a single self-report questionnaire, common method bias (CMB) was assessed following Podsakoff et al. (2003). Procedurally, respondent anonymity was guaranteed, and predictor and criterion variables were separated into distinct questionnaire sections to reduce method-driven response consistency. Statistically, Harman’s single-factor test was conducted by entering all 64 items into an unrotated exploratory factor analysis. The first factor accounted for 40.08% of total variance, well below the 50% threshold (Podsakoff et al., 2003), indicating that CMB does not pose a significant threat to the findings. In SEM analysis, the measurement model defines the relationships between latent constructs and their indicators. Prior to testing the structural model, a confirmatory factor analysis (CFA) was performed to evaluate the measurement model and assess the relationships between latent constructs and their observed indicators. Following the recommendations of methodological scholars, multiple fit indices were utilized to evaluate the adequacy of the measurement model (Hair et al., 2019; Kline, 2023). Table 3 presents the factor loadings and reliability test results for all construct items. The composite reliability (CR) values for all constructs exceeded 0.7, meeting the threshold recommended by Chin (1998) and indicating satisfactory reliability. Additionally, Cronbach’s alpha values for all constructs ranged from 0.963 to 0.967, substantially exceeding the recommended threshold of 0.7, demonstrating excellent internal consistency across all measurement scales. According to Fornell and Larcker (1981), convergent validity is established when factor loadings exceed 0.5, the average variance extracted (AVE) is greater than 0.5, and composite reliability exceeds 0.7. As shown in Table 3, all constructs demonstrated AVE and composite reliability values above these critical thresholds, satisfying the criteria proposed by Fornell and Larcker (1981) and thus confirming adequate convergent validity. To further elaborate on the psychometric properties, all standardized factor loadings exceeded 0.70, indicating that each observed indicator shares substantial variance with its respective latent construct and that item-level reliability (i.e., the squared standardized loading) was consistently above the 0.50 threshold (Hair et al., 2019). Furthermore, the gap between composite reliability and AVE values for each construct was examined to assess whether reliability was inflated by a large number of indicators rather than by strong individual item contributions; across all constructs, the AVE values remained comfortably above 0.50 even when constructs contained four items, confirming that reliability reflects genuine construct-level convergence rather than scale length artifacts (Netemeyer et al., 2003). Collectively, the convergent validity evidence—spanning factor loadings, AVE, composite reliability, and Cronbach’s alpha—provides a comprehensive and multi-criteria assessment that supports the psychometric adequacy of the measurement instrument for all sixteen constructs employed in this study.

Discriminant validity of the measurement model was assessed using the Fornell-Larcker criterion (Fornell and Larcker, 1981). This approach requires that the square root of the average variance extracted (AVE) for each construct exceeds its correlations with all other constructs, thereby confirming that each construct shares more variance with its own indicators than with other constructs in the model. As presented in Table 4, the square root of AVE values for all constructs (bold values on the diagonal) were consistently greater than the corresponding inter-construct correlations (values below the diagonal), thus providing evidence of adequate discriminant validity across all constructs in the measurement model.

**Table 4:** Discriminant validity.

Construct	AA	ACGCI	CCI	CP	EC	IQ	PA	PBC	PE	PI	SN	SOVC	TA	VC	VII	WI
AA	**0.936**	0.338	0.455	0.479	0.412	0.448	0.381	0.491	0.454	0.340	0.406	0.527	0.462	0.413	0.350	0.494
ACGCI	0.352	**0.939**	0.309	0.450	0.332	0.466	0.386	0.472	0.467	0.398	0.474	0.409	0.434	0.379	0.386	0.318
CCI	0.452	0.486	**0.931**	0.405	0.498	0.460	0.412	0.424	0.396	0.423	0.484	0.490	0.458	0.435	0.368	0.467
CP	0.309	0.324	0.409	**0.933**	0.359	0.404	0.403	0.464	0.463	0.366	0.483	0.519	0.484	0.420	0.355	0.402
EC	0.334	0.287	0.362	0.500	**0.938**	0.361	0.298	0.354	0.389	0.522	0.372	0.398	0.333	0.461	0.345	0.339
IQ	0.469	0.453	0.405	0.463	0.364	**0.934**	0.405	0.353	0.514	0.397	0.497	0.518	0.509	0.377	0.365	0.440
PA	0.396	0.294	0.367	0.422	0.525	0.398	**0.933**	0.373	0.471	0.362	0.409	0.404	0.439	0.365	0.409	0.328
PBC	0.294	0.247	0.323	0.338	0.359	0.322	0.292	**0.930**	0.451	0.401	0.435	0.446	0.457	0.404	0.247	0.399
PE	0.469	0.458	0.464	0.398	0.390	0.515	0.392	0.340	**0.935**	0.390	0.491	0.445	0.435	0.515	0.335	0.406
PI	0.386	0.331	0.407	0.413	0.298	0.405	0.364	0.377	0.472	**0.931**	0.459	0.433	0.322	0.413	0.328	0.381
SN	0.409	0.383	0.523	0.489	0.398	0.522	0.434	0.348	0.451	0.407	**0.935**	0.588	0.472	0.475	0.424	0.454
SOVC	0.477	0.314	0.411	0.489	0.382	0.429	0.460	0.292	0.391	0.357	0.593	**0.935**	0.540	0.446	0.411	0.496
TA	0.436	0.351	0.487	0.460	0.333	0.511	0.325	0.463	0.437	0.437	0.543	0.418	**0.933**	0.402	0.271	0.422
VC	0.381	0.330	0.423	0.436	0.459	0.377	0.414	0.409	0.517	0.363	0.448	0.381	0.400	**0.932**	0.344	0.497
VII	0.385	0.355	0.356	0.370	0.347	0.365	0.329	0.247	0.336	0.412	0.415	0.346	0.272	0.344	**0.932**	0.392
WI	0.290	0.452	0.319	0.290	0.250	0.313	0.265	0.294	0.306	0.260	0.340	0.425	0.295	0.270	0.248	**0.928**

AA, audience attitude; ACGCI, anime, comics, and games culture identity; CCI, cross-cultural influence; CP, content professionalism; EC, economic capability; IQ, interaction quality; PA, platform accessibility; PBC, perceived behavioral control; PE, perceived entertainment; PI, peer influence; SN, subjective norm; SOVC, sense of virtual community; TA, time availability; VC, viewing convenience; VII, virtual identity identification; WI, watching intention.

The bottom left corner (including bold numbers) is Fornell-Larcker criterion; the bold values on the diagonal are the square root of AVE for each construct; the top right corner is heterotrait-monotrait ratio (HTMT).

To further strengthen the assessment of discriminant validity, the heterotrait-monotrait ratio of correlations (HTMT) was computed for all construct pairs (Henseler et al., 2015). The HTMT approach has been recommended as a more rigorous criterion than the Fornell-Larcker method, particularly in structural equation modeling contexts where constructs may exhibit similar factor loading magnitudes (Voorhees et al., 2016). As presented in the upper-right portion of Table 4, all HTMT values ranged from 0.247 to 0.588, well below the conservative threshold of 0.85 recommended by Henseler et al. (2015). These results provide robust empirical evidence that each construct captures a distinct theoretical dimension, confirming discriminant validity across the measurement model.

The results indicated that the measurement model demonstrated satisfactory fit to the data. The normed chi-square ratio (χ^2^/df = 2.174) was below the threshold of 3, suggesting acceptable model fit (Kline, 2023). The root mean square error of approximation (RMSEA = 0.058) was below the recommended cutoff of 0.08, indicating that the discrepancy between the hypothesized model and the observed data was within an acceptable range (Browne and Cudeck, 1993; Hu and Bentler, 1999). The standardized root mean square residual (SRMR = 0.020) was substantially lower than the suggested threshold of 0.08, demonstrating minimal residual differences between the model-implied and sample covariance matrices (Hu and Bentler, 1999). Furthermore, the comparative fit index (CFI = 0.923) exceeded the acceptable criterion of 0.90 (Bentler, 1990; Hair et al., 2019), providing additional support for the adequacy of the measurement model. Collectively, these indices suggest that the measurement model exhibited good overall fit, thereby warranting subsequent examination of the structural model and path analysis.

### 4.2 Structural model

Following the establishment of a satisfactory measurement model, the structural model was estimated to test the hypothesized relationships among the latent constructs. Prior to evaluating the structural paths, multicollinearity among the predictor variables was examined through variance inflation factor (VIF) analysis (Hair et al., 2019). VIF values for all structural paths are presented in Table 5. The results indicated that all VIF values ranged from 1.227 (VII → AA) to 1.496 (PE → AA), substantially below the conservative threshold of 3.3 recommended by Diamantopoulos and Siguaw (2006) and the commonly accepted threshold of 5.0 (Hair et al., 2019). These values confirm that multicollinearity does not pose a threat to the stability or interpretability of the structural path estimates in the present model.

**Table 5:** Hypotheses testing result.

Hypothesis	Standardized path coefficient	*z*-value	VIF	Result
H1: Perceived entertainment positively impacts audience attitudes.	0.186***	3.831	1.496	Supported
H2: Virtual identity identification positively impacts audience attitudes.	0.121*	2.469	1.227	supported
H3: Interaction quality positively impacts audience attitudes.	0.201**	3.328	1.446	Supported
H4: Content professionalism positively impacts audience attitudes.	0.275***	4.998	1.360	Supported
H5: Peer influence positively impacts subjective norms.	0.142**	2.651	1.379	Supported
H6: Sense of virtual community positively impacts subjective norms.	0.349***	5.866	1.481	Supported
H7: ACG cultural identity positively impacts subjective norms.	0.219***	3.936	1.281	Supported
H8: Cross-cultural influence positively impacts subjective norms.	0.190**	3.480	1.391	Supported
H9: Viewing convenience positively impacts perceived behavioral control.	0.180**	3.102	1.404	Supported
H10: Economic capability positively impacts perceived behavioral control.	0.139*	2.147	1.302	Supported
H11: Time availability positively impacts perceived behavioral control.	0.280***	4.740	1.348	Supported
H12: Platform accessibility positively impacts perceived behavioral control.	0.148**	3.306	1.296	Supported
H13: Audience attitude positively impacts watching intention.	0.331***	5.753	1.370	Supported
H14: Subjective norm positively impacts watching intention.	0.283***	4.489	1.289	Supported
H15: Perceived behavioral control positively impacts watching intention.	0.129*	2.231	1.405	supported

*p-value < 0.05; **p-value < 0.01; ***p-value < 0.001.

The fit indices for the structural model indicated an acceptable fit to the data. The normed chi-square ratio (χ^2^/df = 2.235) remained below the recommended threshold of 3, the root mean square error of approximation (RMSEA = 0.06) and the standardized root mean square residual (SRMR = 0.06) were both below the cutoff value of 0.08, and the comparative fit index (CFI = 0.928) exceeded the acceptable criterion of 0.90. Collectively, these indices confirmed that the structural model demonstrated adequate overall fit, thereby supporting the subsequent interpretation of path coefficients and hypothesis testing results. The results indicated that watching intention yielded an R^2^ value of 0.308, suggesting that the predictor variables in the model collectively explained 30.8% of the variance in watching intention. Audience attitude demonstrated an R^2^ value of 0.353, indicating that 35.3% of its variance was accounted for by the model. Subjective norms exhibited the highest explanatory power among all endogenous variables, with an R^2^ value of 0.469, signifying that nearly half of the variance was explained by the model. Perceived behavioral control showed an R^2^ value of 0.309, with approximately 30.9% of the variance being explained. According to Cohen (1988) effect size criteria for behavioral science research, R^2^ values of 0.02, 0.13, and 0.26 represent small, medium, and large effect sizes, respectively. The R^2^ values of all endogenous variables in this study ranged from 0.308 to 0.469, exceeding the threshold of 0.26 for large effect sizes, indicating adequate model-level explanatory power. However, it should be noted that R^2^ values reflect the collective contribution of all predictors to each endogenous variable, while the magnitude of individual path coefficients varies considerably across hypothesized relationships, warranting differentiated interpretation in the subsequent discussion.

**Figure 2:**
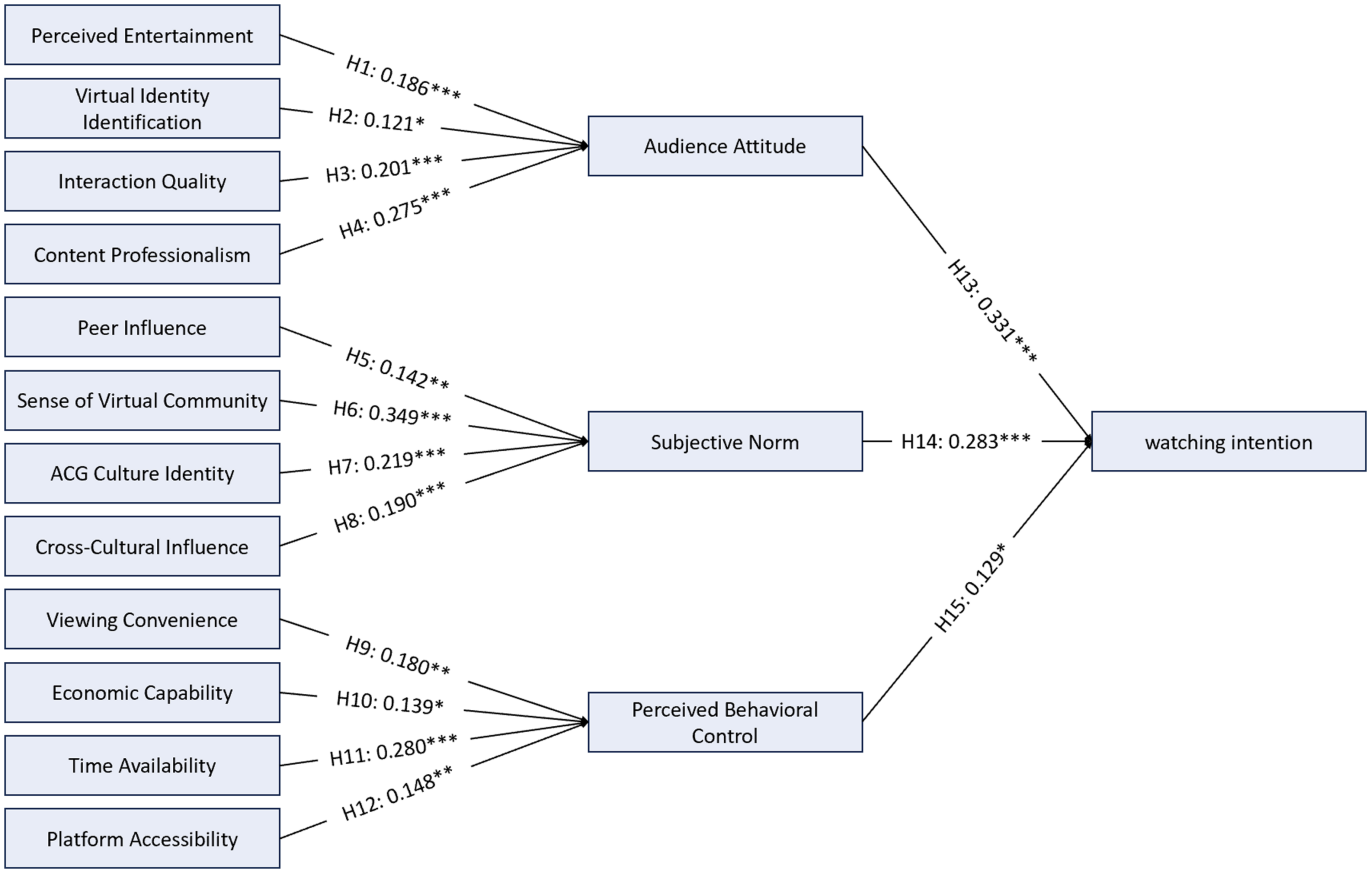
Results of the structural model.

This study proposed 15 path hypotheses, all of which were empirically supported through structural equation modeling analysis. To facilitate interpretation, path coefficients are evaluated with reference to Cohen (2013) benchmarks for standardized regression coefficients in behavioral research, where *β* ≈ 0.10 represents a small effect, *β* ≈ 0.30 a medium effect, and *β* ≈ 0.50 a large effect. Regarding the antecedents of attitude, content professionalism demonstrated the strongest effect (*β* = 0.275, *p* < 0.001), approaching a medium effect size, followed by interaction quality (*β* = 0.201, *p* < 0.01) and perceived entertainment (*β* = 0.186, *p* < 0.001), both representing small-to-medium effects. Virtual identity identification, while statistically significant, exhibited a small effect size (*β* = 0.121, *p* < 0.05), suggesting that its practical influence on audience attitude is relatively limited compared to content- and interaction-related factors. With respect to the determinants of subjective norms, sense of virtual community emerged as the strongest predictor with a medium effect size (*β* = 0.349, *p* < 0.001), followed by ACG cultural identity (*β* = 0.219, *p* < 0.001) and cross-cultural influence (*β* = 0.190, *p* < 0.01), both representing small-to-medium effects. Peer influence exhibited a comparatively small effect (*β* = 0.142, *p* < 0.01). Concerning perceived behavioral control, time availability demonstrated the strongest effect approaching a medium magnitude (*β* = 0.280, *p* < 0.001), while viewing convenience (*β* = 0.180, *p* < 0.01), platform accessibility (*β* = 0.148, *p* < 0.01), and economic capability (*β* = 0.139, *p* < 0.05) all exhibited small effect sizes. Finally, in predicting watching intention, audience attitude demonstrated a medium effect (*β* = 0.331, *p* < 0.001), followed by subjective norms with a small-to-medium effect (*β* = 0.283, *p* < 0.001). Perceived behavioral control, while statistically significant, exhibited a small effect (*β* = 0.129, *p* < 0.05), indicating that its direct practical influence on watching intention is modest relative to the attitudinal and normative pathways Figure 2. This pattern is consistent with prior TPB research in digital entertainment contexts, where perceived behavioral control often demonstrates weaker direct effects on behavioral intention compared to attitude and subjective norms (Ajzen, 2020; Chen and Lin, 2018). The 95% confidence intervals for all path coefficients excluded zero, corroborating the statistical significance of all hypothesized paths, though the practical importance of individual paths varies as noted above.

### 4.3 Indirect effects analysis

To examine the mediating mechanisms underlying VTuber watching intention, specific indirect effects were assessed through bootstrapping procedures with 5,000 resamples, as summarized in Table 6. With respect to the indirect pathways mediated by audience attitude (AA), the results revealed that perceived entertainment (PE → AA → WI, *β* = 0.058, *p* < 0.01), virtual identity identification (VII → AA → WI, *β* = 0.038, *p* < 0.05), interaction quality (IQ → AA → WI, *β* = 0.063, *p* < 0.01), and content professionalism (C*P* → AA → WI, *β* = 0.084, *p* < 0.001) all exerted significant indirect effects on watching intention. Among these, content professionalism demonstrated the strongest indirect influence through attitude. Regarding the indirect pathways mediated by subjective norm (SN), peer influence (PI → SN → WI, *β* = 0.038, *p* < 0.01), sense of virtual community (SOVC → SN → WI, *β* = 0.093, *p* < 0.001), ACG culture identity (ACGCI → SN → WI, *β* = 0.050, *p* < 0.001), and cross-cultural influence (CCI → SN → WI, *β* = 0.059, *p* < 0.001) were all found to exert significant indirect effects on watching intention, with sense of virtual community yielding the most pronounced indirect effect. As for the indirect pathways mediated by perceived behavioral control (PBC), viewing convenience (VC → PBC → WI, *β* = 0.021, *p* < 0.05), economic capability (EC → PBC → WI, *β* = 0.016, *p* < 0.05), time availability (TA → PBC → WI, *β* = 0.034, *p* < 0.05), and platform accessibility (PA → PBC → WI, *β* = 0.018, *p* < 0.05) all demonstrated statistically significant indirect effects on watching intention. However, it is worth noting that the indirect effects through PBC are notably smaller in magnitude than those through attitude and subjective norms, reflecting the modest direct effect of PBC on watching intention (*β* = 0.129). The 95% bias-corrected confidence intervals for all indirect effects excluded zero, confirming statistical significance across all mediating pathways, though the practical importance of the PBC-mediated pathways is comparatively limited.

**Table 6:** Specific indirect effects.

Indirect path	Indirect effect	Standard error	*z*-value	Confidence interval
PE - > AA - > WI	0.058**	0.021	2.762	(0.021, 0.108)
VII - > AA - > WI	0.038*	0.018	2.111	(0.005, 0.081)
IQ - > AA - > WI	0.063**	0.022	2.864	(0.026, 0.115)
CP - > AA - > WI	0.084***	0.022	3.818	(0.045, 0.139)
PI - > SN - > WI	0.038**	0.016	2.375	(0.010, 0.076)
SOVC - > SN - > WI	0.093***	0.023	4.043	(0.052, 0.147)
ACGCI - > SN - > WI	0.050***	0.017	2.941	(0.022, 0.091)
CCI - > SN - > WI	0.059***	0.016	3.688	(0.031, 0.099)
VC - > PBC - > WI	0.021*	0.013	1.615	(0.002, 0.056)
EC - > PBC - > WI	0.016*	0.010	1.600	(0.002, 0.045)
TA - > PBC - > WI	0.034*	0.017	2.000	(0.005, 0.076)
PA - > PBC - > WI	0.018*	0.011	1.636	(0.002, 0.049)

*p-value < 0.05; **p-value < 0.01; ***p-value < 0.001.

## 5 Conclusion

This section synthesizes the quantitative findings reported in Section 4 with the theoretical framework developed in Section 2 to derive both practical and theoretical implications. The integration of statistical evidence (path coefficients, variance explained, mediation effects) with the conceptual logic underlying each hypothesized relationship is central to the discussion that follows. Specifically, the discussion explicitly connects the relative magnitude and statistical significance of each path coefficient to the corresponding theoretical arguments, enabling a nuanced assessment of which theoretical mechanisms receive strong empirical support and which reveal unexpected boundary conditions. This integrative approach—linking the quantitative structural model results with the theoretical rationale for each decomposed antecedent—ensures that the conclusions are grounded simultaneously in empirical evidence and theoretical reasoning, thereby strengthening the overall methodological rigor and interpretive coherence of the study (Creswell and Plano Clark, 2018).

### 5.1 Practical implications

The findings demonstrate that audience attitude and subjective norms exert strong and significant positive influences on VTuber watching intention, while perceived behavioral control shows relatively weaker direct effects. These results align with existing livestreaming research: Chen and Lin (2018) confirmed that entertainment perception and interaction experience strengthen viewing attitudes, while Zhao et al. (2018) highlighted how subjective norms formed through community interaction and social influence enhance audience participation intention. The comprehensive mediation analysis further reveals that content professionalism emerges as the strongest indirect pathway to watching intention through audience attitude, while sense of virtual community demonstrates the most significant indirect effect through subjective norms. Thus, this study validates the central role of audience attitude and subjective norms in VTuber viewing behavior while identifying specific strategic priorities. From a practical perspective, VTuber platform operators should prioritize content professionalism enhancement and community building initiatives as primary strategic focuses, followed by entertainment value and interactivity improvements to reinforce positive viewing attitudes and strengthen perceived subjective norms.

The research reveals that content professionalism demonstrates the strongest influence on audience attitude, followed by interaction quality and perceived entertainment, while virtual identity identification shows a relatively weak effect. This hierarchy of importance aligns with existing literature: Hu et al. (2017) noted that audiences prioritize content quality and interactive experiences, while the relatively weaker role of virtual identity identification suggests that audiences view VTubers primarily as content creators rather than identity extensions. From practical perspectives, VTuber operators should implement comprehensive content quality enhancement programs, including professional training for streamers, content planning and preparation protocols, and quality assessment frameworks. Emphasis should be placed on developing specialized expertise and distinctive content styles that meet specific audience needs. Interactive quality improvement should focus on response timeliness, engagement frequency, and personalized attention to audience feedback. While virtual identity development remains important for brand differentiation, it should be positioned as a supplementary strategy rather than the primary operational focus.

The study reveals that sense of virtual community exerts the strongest influence on subjective norms, followed by ACG culture identity and cross-cultural influence, while peer influence demonstrates moderate effects. These findings align with existing literature: Hu et al. (2017) confirmed that virtual community belonging significantly promotes - continued participation, while the significant role of ACG culture identity reflects the growing mainstream acceptance of anime gaming culture through VTuber mediation. The substantial cross-cultural influence effect suggests that Chinese VTuber audiences perceive VTubers as meaningful conduits of cross-cultural exposure, indicating their potential value as cultural mediators within the Bilibili ecosystem. This includes establishing dedicated community spaces, organizing regular ACG-themed events, facilitating fan-generated content creation, and developing community recognition and reward systems. Cross-cultural exchange should be actively promoted through multilingual support, cultural festival celebrations, and international collaboration events. Platforms should design features that highlight cultural diversity and encourage intercultural dialogue among audiences from different backgrounds.

The research indicates that time availability exerts the strongest influence on perceived behavioral control, substantially exceeding the effects of viewing convenience, economic capability, and platform accessibility. This pattern reflects the dominance of attention economy over traditional resource constraints in contemporary digital content consumption. The finding that time availability supersedes economic capability as the primary constraining factor aligns with the broader trend toward content accessibility and platform proliferation. For practical and managerial implications, VTuber platforms should prioritize temporal flexibility optimization through on-demand viewing options, content fragmentation strategies, and personalized scheduling features. Mobile-first design approaches should accommodate fragmented viewing patterns, while intelligent recommendation systems should optimize content discovery efficiency to maximize time utility. Although economic capability shows relatively weaker influence, platforms should maintain tiered engagement options to ensure accessibility across diverse economic backgrounds. Platform accessibility improvements should focus on reducing cognitive load and operational complexity, while technical infrastructure optimization should ensure seamless cross-device experiences that minimize time investment barriers.

### 5.2 Theoretical contributions

This study contextualizes and systematically applies the decomposed Theory of Planned Behavior (DTPB; Taylor and Todd, 1995) within VTuber consumption contexts, yielding three significant theoretical contributions that directly address the declared research gaps in understanding VTuber audience behavioral mechanisms. Collectively, these contributions move beyond incremental extension of TPB to a new context; rather, they fundamentally challenge established assumptions about how audiences engage with hybrid human-virtual content creators, revealing that the psychological and cultural mechanisms governing VTuber consumption diverge from those documented in adjacent domains such as livestreaming and virtual influencer research. The empirical evidence presented here offers a recalibrated theoretical lens for understanding audience behavior in an era of increasingly avatar-mediated digital entertainment.

The first theoretical contribution lies in the comprehensive, context-specific decomposition of TPB dimensions for VTuber audience research. Addressing the first research gap regarding the absence of comprehensive frameworks integrating VTuber-specific factors, this study systematically decomposes attitude, subjective norms, and perceived behavioral control into twelve domain-specific antecedent variables. While nine of these variables—perceived entertainment, interaction quality, content professionalism, peer influence, sense of virtual community, viewing convenience, economic capability, time availability, and platform accessibility—represent established constructs recontextualized from livestreaming and digital consumer behavior literature, three variables—virtual identity identification, ACG culture identity, and cross-cultural influence—are conceptually novel constructs that capture distinctive features of VTuber consumption absent from prior DTPB applications. This differentiated integration demonstrates that VTuber audience behavior involves mechanisms that cannot be fully accounted for by existing livestreaming or virtual influencer frameworks, justifying context-specific theoretical treatment. Critically, the quantitative results substantiate this framework’s theoretical value: the structural model explained 46.9% of the variance in subjective norms, 35.3% in attitude, and 30.9% in perceived behavioral control, indicating that the decomposed antecedent structure provides substantial predictive power (Cohen, 2013). Moreover, the three domain-specific novel constructs—virtual identity identification (*β* = 0.121), ACG culture identity (*β* = 0.219), and cross-cultural influence (*β* = 0.190)—all demonstrated statistically significant effects, empirically confirming that these VTuber-specific dimensions capture variance that would be missed by generic livestreaming or virtual influencer models. This integration of quantitative evidence with theoretical argumentation provides empirical grounding for the claim that VTuber consumption requires a distinct DTPB configuration, moving beyond speculative theorization to evidence-based framework development.

The second theoretical contribution centers on the identification of boundary conditions that differentiate VTuber consumption from traditional livestreaming and influencer engagement. The empirical findings reveal two notable boundary conditions. First, within the attitude dimension, content professionalism emerges as the strongest predictor while virtual identity identification shows relatively weaker influence. This counterintuitive finding challenges the theoretical expectation—derived from virtual influencer and avatar research (Jin, 2012; Ratan and Dawson, 2016)—that identification with virtual personas would be central to VTuber engagement. Instead, the results indicate that audiences approach VTubers primarily as professional content creators rather than as identity extensions, suggesting that the human agency behind VTuber personas may render virtual identity less psychologically distinct than theoretically assumed. Second, within the perceived behavioral control dimension, time availability substantially exceeds economic capability, viewing convenience, and platform accessibility as the primary constraining factor. This pattern diverges from traditional media consumption models where economic resources typically dominate behavioral constraints, providing empirical evidence for the primacy of attention economy dynamics in contemporary virtual entertainment consumption. These boundary conditions collectively reveal that established antecedent hierarchies from livestreaming research do not directly transfer to VTuber contexts, demonstrating the value of context-specific DTPB applications. Meanwhile, addressing the second research gap concerning cross-cultural influence mechanisms, this study contributes to cross-cultural consumer behavior theory by demonstrating that perceived exposure to cross-cultural content through VTubers significantly shapes subjective norms among Chinese audiences. While this finding is derived from a culturally homogeneous sample and thus reflects perceived cross-cultural influence rather than observed cross-cultural differences, it nonetheless reveals the normative impact of cross-cultural content exposure within a single-country context. The research introduces cross-cultural influence and ACG culture identity as significant predictors of subjective norms—constructs that are conceptually distinct from the interpersonal and community-based normative influences typically examined in livestreaming research. Cross-cultural influence captures transnational cultural flows mediated through anime-aesthetic VTuber content, while ACG culture identity reflects subcultural normative pressures that operate through shared identification with a specific cultural community rather than through direct interpersonal reference groups. The significant effects of both constructs reveal VTubers’ unique value as cultural mediators, facilitating the formation of globalized virtual cultural communities that transcend traditional geographic and linguistic boundaries, and contributing novel insights into how global virtual content shapes local consumption patterns and norm formation processes.

The third theoretical contribution involves the methodological demonstration of DTPB’s applicability in emerging virtual entertainment domains. By successfully applying the decomposition approach to a context substantially different from DTPB’s original technology adoption focus, this study expands the range of behavioral domains where DTPB provides effective predictive frameworks. The structured decomposition—distinguishing content/interaction factors (attitude), community/context factors (subjective norms), and platform/resource factors (perceived behavioral control)—offers a replicable analytical template for future research on audience behavior in other hybrid human-virtual entertainment contexts, such as AI-generated content creators, metaverse performers, or virtual reality streamers. Importantly, the acceptable-to-good model fit indices (CFI = 0.928, RMSEA = 0.06, SRMR = 0.06) and the statistical significance of all fifteen hypothesized paths provide robust methodological evidence that the DTPB decomposition approach retains its structural coherence when transplanted from technology adoption to virtual entertainment. This finding has direct implications for the broader TPB/DTPB literature, as it suggests that the decomposition logic is theoretically portable across behavioral domains, provided that domain-specific antecedents are carefully theorized and operationalized. In doing so, this study responds to Ajzen’s (2020) call for contextually enriched applications of TPB that move beyond generic replications to yield substantive theoretical insights about domain-specific behavioral mechanisms.

### 5.3 Research limitations and future works

Despite its contributions, several limitations should be acknowledged. First, the cross-sectional design precludes causal inferences; the observed directional relationships should be interpreted as theoretically informed associations rather than confirmed causal effects (Kline, 2023). Future research should employ longitudinal designs to establish temporal precedence and capture the dynamic evolution of viewing behavior.

Second, the sample was drawn exclusively from Chinese Bilibili users recruited through WeChat groups, constituting a convenience sampling approach that may introduce self-selection bias toward more committed audience segments (Wright, 2005). The single-country sampling also limits external validity, and the cross-cultural influence (CCI) construct specifically captures perceived cultural exposure within a culturally homogeneous sample rather than observed cross-cultural differences. Future research should employ multi-country comparative designs across platforms such as Twitch and YouTube to examine whether the identified relationships replicate in different cultural contexts (Chen and Lin, 2018).

Third, all constructs were measured through self-report instruments, which are susceptible to social desirability bias and common method variance. Although procedural and statistical safeguards were implemented (Podsakoff et al., 2003), shared method variance cannot be entirely ruled out. Future research could combine self-report data with behavioral trace data (e.g., viewing duration, engagement logs, donation records) to reduce method-related concerns.

Fourth, this study examines watching intention rather than actual viewing behavior. Meta-analytic evidence suggests that the intention-behavior gap can be substantial in digital entertainment contexts (Sheeran and Webb, 2016), and the present findings reflect motivational readiness rather than actual consumption patterns. Incorporating objective behavioral outcomes in future research would strengthen the practical validity of the model.

Fifth, the current model may not incorporate all relevant influencing factors, such as parasocial relationship strength, streamer charisma, or viewer personality traits (Hu et al., 2017). Future research could integrate these variables and employ qualitative approaches to provide richer insights into the psychological mechanisms underlying VTuber audience engagement (Lee and Watkins, 2016).

Sixth, although the sample size of 352 meets multiple established thresholds for CB-SEM analysis (Kline, 2023; Westland, 2010; Wolf et al., 2013), it remains moderate relative to the complexity of the proposed model with 16 latent variables and 64 observed indicators. While the subject-to-variable ratio of 5.5:1 satisfies conventional minimums, achieving higher ratios (e.g., 10:1 or above) would improve the stability of parameter estimates and increase statistical power for detecting smaller effects (Hair et al., 2019). Future research should aim for larger and more demographically diverse samples to enhance the robustness and generalizability of the structural model, and to enable more fine-grained analyses such as multi-group comparisons across demographic subgroups or platform usage intensity levels.

## Data Availability

The raw data supporting the conclusions of this article will be made available by the authors, without undue reservation.

## References

[ref1] Ajzen I. (1991). The theory of planned behavior. Organ. Behav. Hum. Decis. Process. 50, 179–211. doi: 10.1016/0749-5978(91)90020-T

[ref2] Ajzen I. (2002). Perceived behavioral control, self-efficacy, locus of control, and the theory of planned behavior. J. Appl. Soc. Psychol. 32, 665–683. doi: 10.1111/j.1559-1816.2002.tb00236.x

[ref3] Ajzen I. (2020). The theory of planned behavior: frequently asked questions. Human Behav. Emerging Technol. 2, 314–324. doi: 10.1002/hbe2.195

[ref4] Anderson J. C. Gerbing D. W. (1988). Structural equation modeling in practice: a review and recommended two-step approach. Psychol. Bull. 103, 411–423. doi: 10.1037/0033-2909.103.3.411

[ref5] Appiah O. (2004). Effects of ethnic identification on web browsers' attitudes toward and navigational patterns on race-targeted sites. Commun. Res. 31, 312–337. doi: 10.1177/0093650203261515

[ref9003] Armitage C. J. Conner M. (2001). Efficacy of the theory of planned behaviour: A meta-analytic review. British Journal of Social Psychology, 40, 471–499. 10.1348/014466601164939 11795063

[ref6] Ashforth B. E. Mael F. (1989). Social identity theory and the organization. Acad. Manag. Rev. 14, 20–39. doi: 10.5465/amr.1989.4278999

[ref7] Baltar F. Brunet I. (2012). Social research 2.0: virtual snowball sampling method using Facebook. Internet Res. 22, 57–74. doi: 10.1108/10662241211199960

[ref9006] Bandura A. (1982). Self-efficacy mechanism in human agency. American Psychologist, 37, 122–147. 10.1037/0003-066X.37.2.122

[ref8] Belanche D. Flavián M. Pérez-Rueda A. (2020). Influencers on Instagram: antecedents and consequences of opinion leadership. J. Bus. Res. 117, 510–519. doi: 10.1016/j.jbusres.2018.07.005

[ref9014] Bentler P. M. (1990). Comparative fit indexes in structural models. Psychological Bulletin, 107, 238–246. 2320703 10.1037/0033-2909.107.2.238

[ref9] Bentler P. M. Chou C.-P. (1987). Practical issues in structural modeling. Sociol. Methods Res. 16, 78–117. doi: 10.1177/0049124187016001004

[ref10] Berry J. W. (1997). Immigration, acculturation, and adaptation. Appl. Psychol. 46, 5–34. doi: 10.1111/j.1464-0597.1997.tb01087.x

[ref11] Blanchard A. L. Markus M. L. (2004). The experienced "sense" of a virtual community: characteristics and processes. ACM SIGMIS Database Database Adv. Inf. Syst. 35, 64–79. doi: 10.1145/968464.968470

[ref12] Brislin R. W. (1970). Back-translation for cross-cultural research. J. Cross-Cult. Psychol. 1, 185–216. doi: 10.1177/135910457000100301

[ref9012] Browne M. W. Cudeck R. (1993). Alternative ways of assessing model fit. In Bollen K. A. Long J. S. (Eds.), Testing structural equation models (pp. 136–162). Sage Publications. Newbury Park, CA.

[ref13] Chen C. C. Lin Y. C. (2018). What drives live-stream usage intention? The perspectives of flow, entertainment, social interaction, and endorsement. Telemat. Inform. 35, 293–303. doi: 10.1016/j.tele.2017.12.003

[ref14] Cheung C. M. Lee M. K. (2010). A theoretical model of intentional social action in online social networks. Decis. Support. Syst. 49, 24–30. doi: 10.1016/j.dss.2009.12.006

[ref9011] Chin W. W. (1998). The partial least squares approach to structural equation modeling. Modern Methods for Business Research, 295, 295–336.

[ref16] Cohen E. L. Tyler W. J. (2016). Examining perceived distance and personal authenticity as mediators of the effects of ghost-tweeting on parasocial relationships and fan evaluations. Cyberpsychol. Behav. Soc. Netw. 19, 342–346. doi: 10.1089/cyber.2015.0657, 27186899

[ref15] Cohen J. (2013). Statistical Power Analysis for the Behavioral Sciences. New York: Routledge.

[ref9010] Cohen J. (1988). Statistical power analysis for the behavioral sciences (2nd ed.). Hillsdale, NJ: Lawrence Erlbaum Associates. 10.4324/9780203771587

[ref17] Creswell J. W. Plano Clark V. L. (2018). Designing and Conducting Mixed Methods Research. 3rd Edn. CA: Sage Publications.

[ref9009] Dabchick B. G. Fidell L. S. (2013). Using multivariate statistics (6th ed.). Pearson, Upper Saddle River, NJ.

[ref20] Davis F. D. (1989). Perceived usefulness, perceived ease of use, and user acceptance of information technology. MIS Q. 13, 319–340. doi: 10.2307/249008

[ref9007] DeSimone J. A. Harms P. D. DeSimone A. J. (2015). Best practice recommendations for data screening. Journal of Organizational Behavior, 36, 171–181. 10.1002/job.1962

[ref22] Deutsch M. Gerard H. B. (1955). A study of normative and informational social influences upon individual judgment. J. Abnorm. Soc. Psychol. 51, 629–636. doi: 10.1037/h0046408, 13286010

[ref23] Dholakia U. M. Bagozzi R. P. Pearo L. K. (2004). A social influence model of consumer participation in network- and small-group-based virtual communities. Int. J. Res. Mark. 21, 241–263. doi: 10.1016/j.ijresmar.2003.12.004

[ref9008] Diamantopoulos A. Siguaw J. A. (2006). Formative versus reflective indicators in organizational measure development: A comparison and empirical illustration. British Journal of Management, 17, 263–282. 10.1111/j.1467-8551.2006.00500.x

[ref25] Fishbein M. Ajzen I. (1977). Belief, attitude, intention, and behavior: an introduction to theory and research. Philos. Rhetor. 10, 130–132.

[ref26] Fornell C. Larcker D. F. (1981). Evaluating structural equation models with unobservable variables and measurement error. J. Mark. Res. 18, 39–50. doi: 10.1177/002224378101800104

[ref27] Gefen D. Straub D. W. (2000). The relative importance of perceived ease of use in IS adoption: a study of e-commerce adoption. J. Assoc. Inf. Syst. 1:8. doi: 10.17705/1jais.00008

[ref9004] Hagger M. S. Chatzisarantis N. L. D. Biddle S. J. H. (2002). A meta-analytic review of the theories of reasoned action and planned behavior in physical activity: Predictive validity and the contribution of additional variables. Journal of Sport \u0026amp; Exercise Psychology, 24, 3–32. 10.1123/jsep.24.1.3

[ref28] Hair J. F. Black W. C. Babin B. J. Anderson R. E. (2019). Multivariate data analysis. 8th Edn. Andover, Hampshire, UK: Cengage Learning.

[ref29] Hair J. F. Hult G. T. M. Ringle C. M. Sarstedt M. (2022). A Primer on Partial Least Squares Structural Equation Modeling (PLS-SEM). 3rd Edn. CA: Sage Publications.

[ref31] Hamilton W. A. Garretson O. Kerne A. (2014). “Streaming on twitch: fostering participatory communities of play within live mixed media,” in Proceedings of the SIGCHI Conference on Human Factors in Computing Systems, New York: Association for Computing Machinery. 1315–1324. doi: 10.1145/2556288.2557048

[ref33] Henseler J. Ringle C. M. Sarstedt M. (2015). A new criterion for assessing discriminant validity in variance-based structural equation modeling. J. Acad. Mark. Sci. 43, 115–135. doi: 10.1007/s11747-014-0403-8

[ref34] Hilvert-Bruce Z. Neill J. T. Sjöblom M. Hamari J. (2018). Social motivations of live-streaming viewer engagement on twitch. Comput. Hum. Behav. 84, 58–67. doi: 10.1016/j.chb.2018.02.013

[ref36] Hofstede G. (1984). Cultural dimensions in management and planning. Asia Pac. J. Manag. 1, 81–99. doi: 10.1007/BF01733682

[ref37] Hollebeek L. D. Macky K. (2019). Digital content marketing’s role in fostering consumer engagement, trust, and value: framework, fundamental propositions, and implications. J. Interact. Mark. 45, 27–41. doi: 10.1016/j.intmar.2018.07.003

[ref38] Homburg C. Koschate N. Hoyer W. D. (2005). Do satisfied customers really pay more? A study of the relationship between customer satisfaction and willingness to pay. J. Mark. 69, 84–96. doi: 10.1509/jmkg.69.2.84.60760

[ref40] Hu L. Min Q. Han S. Liu Z. (2020). Understanding followers' stickiness to digital influencers: the effect of psychological responses. Int. J. Inf. Manag. 54:102169. doi: 10.1016/j.ijinfomgt.2020.102169

[ref9013] Hu L. T. Bentler P. M. (1999). Cutoff criteria for fit indexes in covariance structure analysis: Conventional criteria versus new alternatives. Structural Equation Modeling: A Multidisciplinary Journal, 6, 1–55. 10.1080/10705519909540118

[ref41] Hu M. Zhang M. Wang Y. (2017). Why do audiences choose to keep watching on live video streaming platforms? An explanation of dual identification framework. Comput. Hum. Behav. 75, 594–606. doi: 10.1016/j.chb.2017.06.006

[ref43] Jin S. A. A. (2012). The virtual malleable self and the virtual identity discrepancy model: investigative frameworks for virtual possible selves and others in avatar-based identity construction and social interaction. Comput. Human Behav. 28, 2160–2168. doi: 10.1016/j.chb.2012.06.022

[ref44] Jin S. A. A. Martin C. (2015). “A match made…Online?” the effects of user-generated online dater profile types (free-spirited versus uptight) on other users' perception of trustworthiness, interpersonal attraction, and personality. Cyberpsychol. Behav. Soc. Netw. 18, 320–327. doi: 10.1089/cyber.2014.0564 26075918

[ref45] Jung J. Lin W. Y. Kim Y. C. (2012). The dynamic relationship between east Asian adolescents' use of the internet and their use of other media. New Media Soc. 14, 969–986. doi: 10.1177/1461444812437516

[ref48] Kim Y. Sohn D. Choi S. M. (2011). Cultural difference in motivations for using social network sites: a comparative study of American and Korean college students. Comput. Human Behav. 27, 365–372. doi: 10.1016/j.chb.2010.08.015

[ref49] Kline R. B. (2023). Principles and Practice of Structural Equation Modeling. 5th Edn. New York: Guilford Press.

[ref50] Koh J. Kim Y. G. (2004). Knowledge sharing in virtual communities: an e-business perspective. Expert Syst. Appl. 26, 155–166. doi: 10.1016/S0957-4174(03)00116-7

[ref54] Lee J. E. Watkins B. (2016). YouTube vloggers' influence on consumer luxury brand perceptions and intentions. J. Bus. Res. 69, 5753–5760. doi: 10.1016/j.jbusres.2016.04.171

[ref55] Li L. Zhou W. Chen Y. (2024). Factors influencing users’ watching intention in virtual streaming: the perspective of flow experience. Kybernetes 55, 1–22. doi: 10.1108/K-03-2024-0721

[ref57] Lim J. S. Choe M. J. Zhang J. Noh G. Y. (2020). The role of wishful identification, emotional engagement, and parasocial relationships in repeated viewing of live-streaming games: a social cognitive theory perspective. Comput. Human Behav. 108:106327. doi: 10.1016/j.chb.2020.106327

[ref58] Lin K. Y. Lu H. P. (2011). Why people use social networking sites: an empirical study integrating network externalities and motivation theory. Comput. Human Behav. 27, 1152–1161. doi: 10.1016/j.chb.2010.12.009

[ref59] Liu D. Wright K. B. Hu B. (2018). A meta-analysis of social network site use and social support. Comput. Educ. 127, 201–213. doi: 10.1016/j.compedu.2018.08.024

[ref63] Luo M. M. Chen J. S. Ching R. K. Liu C. C. (2011). An examination of the effects of virtual experiential marketing on online customer intentions and loyalty. Serv. Ind. J. 31, 2163–2191. doi: 10.1080/02642069.2010.503885

[ref61] Lu Z. Shen C. Li J. Shen H. Wigdor D. (2021). “More kawaii than a real-person streamer: understanding how the otaku community engages with virtual YouTubers,” in Proceedings of the 2021 CHI Conference on Human Factors in Computing Systems, (New York: Association for Computing Machinery), 1–14. doi: 10.1145/3411764.3445660

[ref62] Lu Z. Xia H. Heo S. Wigdor D. (2018). “You watch, you give, and you engage: a study of live streaming practices in China,” in Proceedings of the 2018 CHI Conference on Human Factors in Computing Systems, (New York: Association for Computing Machinery), 1–13. doi: 10.1145/3173574.3174040

[ref65] Markus H. R. Kitayama S. (1991). Culture and the self: implications for cognition, emotion, and motivation. Psychol. Rev. 98, 224–253. doi: 10.1037/0033-295X.98.2.224

[ref66] Maruyama M. Robertson S. P. Douglas S. Raine R. (2017). “Social watching a civic broadcast: understanding the effects of positive feedback and other users' opinions,” in Proceedings of the 2017 ACM Conference on Computer Supported Cooperative Work and Social Computing, (New York: Association for Computing Machinery), 794–807. doi: 10.1145/2998181.2998340

[ref9002] Meade A. W. Craig S. B. (2012). Identifying careless responses in survey data. Psychological Methods, 17, 437–456. 10.1037/a0028085 22506584

[ref68] Netemeyer R. G. Bearden W. O. Sharma S. (2003). Scaling Procedures: Issues and Applications. CA: Sage Publications.

[ref69] Oh C. S. Bailenson J. N. Welch G. F. (2018). A systematic review of social presence: definition, antecedents, and implications. Front. Robotics AI 5:114. doi: 10.3389/frobt.2018.00114, 33500993 PMC7805699

[ref71] Park H. J. Lin L. M. (2020). The effects of match-ups on the consumer attitudes toward internet celebrities and their live streaming contents in the context of product endorsement. J. Retail. Consum. Serv. 52:101934. doi: 10.1016/j.jretconser.2019.101934

[ref72] Park H. S. Smith S. W. (2017). Distinctiveness and influence of subjective norms, personal descriptive and injunctive norms, and societal descriptive and injunctive norms on behavioral intent: a case of two behaviors critical to organ donation. Hum. Commun. Res. 33, 194–218. doi: 10.1111/j.1468-2958.2007.00296.x

[ref73] Pavlou P. A. Fygenson M. (2006). Understanding and predicting electronic commerce adoption: an extension of the theory of planned behavior. MIS Q. 30, 115–143. doi: 10.2307/25148720

[ref75] Podsakoff P. M. MacKenzie S. B. Lee J. Y. Podsakoff N. P. (2003). Common method biases in behavioral research: a critical review of the literature and recommended remedies. J. Appl. Psychol. 88, 879–903. doi: 10.1037/0021-9010.88.5.879, 14516251

[ref76] Ratan R. Dawson M. (2016). When mii is me: a psychophysiological examination of avatar self-relevance. Commun. Res. 43, 1065–1093. doi: 10.1177/0093650215570652

[ref9001] Ringle C. M. Wende S. Becker J.-M. (2024). SmartPLS 4. Bönningstedt: SmartPLS. Available online at: https://www.smartpls.com

[ref9005] Sheeran P. Webb T. L. (2016). The intention–behavior gap. Social and Personality Psychology Compass, 10, 503–518. 10.1111/spc3.12265

[ref78] Shen K. N. Khalifa M. (2008). Exploring multidimensional conceptualization of social presence in the context of online communities. Int. J. Hum. Comp. Interact. 24, 722–748. doi: 10.1080/10447310802335789

[ref79] Sherry J. L. (2004). Flow and media enjoyment. Commun. Theory 14, 328–347. doi: 10.1111/j.1468-2885.2004.tb00318.x

[ref80] Sjöblom M. Hamari J. (2017). Why do people watch others play video games? An empirical study on the motivations of twitch users. Comput. Human Behav. 75, 985–996. doi: 10.1016/j.chb.2016.10.019

[ref81] Sjöblom M. Törhönen M. Hamari J. Macey J. (2017). Content structure is king: an empirical study on gratifications, game genres and content type on twitch. Comput. Human Behav. 73, 161–171. doi: 10.1016/j.chb.2017.03.036

[ref82] Sogari G. Corbo C. Macconi M. Menozzi D. Mora C. (2015). Consumer attitude towards sustainable-labelled wine: an exploratory approach. Int. J. Wine Bus. Res. 27, 312–328. doi: 10.1108/IJWBR-12-2014-0053

[ref83] Soper D. S.. (2024). A-priori sample size calculator for structural equation models. Available online at: https://www.danielsoper.com/statcalc

[ref84] Stein J. P. Breves P. L. Anders N. (2024). Parasocial interactions with real and virtual influencers: the role of perceived similarity and human-likeness. New Media Soc. 26, 3433–3453. doi: 10.1177/14614448221102900

[ref85] Sun H. Zhang P. (2006). The role of moderating factors in user technology acceptance. Int. J. Hum.-Comput. Stud. 64, 53–78. doi: 10.1016/j.ijhcs.2005.04.013

[ref9050] Tabachnick B. G. Fidell L. S. (2013). Using multivariate statistics (6th ed.). Upper Saddle River, NJ: Pearson.

[ref86] Tajfel H. (1982). Social psychology of intergroup relations. Annu. Rev. Psychol. 33, 1–39. doi: 10.1146/annurev.ps.33.020182.000245

[ref87] Tang Q. Liu L. Liu Z. (2025). Understanding how the Chinese youth engages with virtual streamers in digital era: an approach of affordance. Int. Commun. Chin. Cult. 12, 17–35. doi: 10.1007/s40636-025-00318-1

[ref88] Taufik D. Bolderdijk J. W. Steg L. (2016). Going green? The relative importance of feelings over calculation in driving environmental intent in the Netherlands and the United States. Energy Res. Soc. Sci. 22, 52–62. doi: 10.1016/j.erss.2016.08.012

[ref89] Taylor S. Todd P. A. (1995). Understanding information technology usage: a test of competing models. Inf. Syst. Res. 6, 144–176. doi: 10.1287/isre.6.2.144

[ref91] Valentine K. D. Kopcha T. J. Vagle M. D. (2019). Phenomenological methodologies in the field of educational communications and technology. TechTrends 63, 525–535. doi: 10.1007/s11528-018-0317-2

[ref92] Venkatesh V. Morris M. G. Davis G. B. Davis F. D. (2003). User acceptance of information technology: toward a unified view. MIS Q. 27, 425–478. doi: 10.2307/30036540

[ref93] Venkatesh V. Thong J. Y. Xu X. (2012). Consumer acceptance and use of information technology: extending the unified theory of acceptance and use of technology. MIS Q. 36, 157–178. doi: 10.2307/41410412

[ref94] Voorhees C. M. Brady M. K. Calantone R. Ramirez E. (2016). Discriminant validity testing in marketing: an analysis, causes for concern, and proposed remedies. J. Acad. Mark. Sci. 44, 119–134. doi: 10.1007/s11747-015-0455-4

[ref95] Vorderer P. Klimmt C. Ritterfeld U. (2004). Enjoyment: at the heart of media entertainment. Commun. Theory 14, 388–408. doi: 10.1111/j.1468-2885.2004.tb00321.x

[ref96] Wan C. Chiou W. (2016). Why are adolescents addicted to online gaming? An interview study in Taiwan. Cyberpsychol. Behav. 9, 762–766. doi: 10.1089/cpb.2006.9.762, 17201603

[ref98] Wang X. Yu C. Wei Y. (2012). Social media peer communication and impacts on purchase intentions: a consumer socialization framework. J. Interact. Mark. 26, 198–208. doi: 10.1016/j.intmar.2011.11.004

[ref97] Wan Q. Lu Z. (2024). Investigating VTubing as a reconstruction of streamer self-presentation: identity, performance, and gender. Proceed. ACM Hum. Comp. Interact. 8, 1–22. doi: 10.1145/3637357

[ref99] Westland J. C. (2010). Lower bounds on sample size in structural equation modeling. Electron. Commer. Res. Appl. 9, 476–487. doi: 10.1016/j.elerap.2010.07.003

[ref100] Wohn D. Y. Freeman G. McLaughlin C. (2018). “Explaining viewers' emotional, instrumental, and financial support provision for live streamers,” in Proceedings of the 2018 CHI Conference on human Factors in Computing Systems, (New York: Association for Computing Machinery), 1–13. doi: 10.1145/3173574.3174048

[ref101] Wolf E. J. Harrington K. M. Clark S. L. Miller M. W. (2013). Sample size requirements for structural equation models: an evaluation of power, bias, and solution propriety. Educ. Psychol. Meas. 73, 913–934. doi: 10.1177/0013164413495237, 25705052 PMC4334479

[ref102] Wongkitrungrueng A. Assarut N. (2018). The role of live streaming in building consumer trust and engagement with social commerce sellers. J. Bus. Res. 117, 543–556. doi: 10.1016/j.jbusres.2018.08.032

[ref105] Wright K. B. (2005). Researching internet-based populations: advantages and disadvantages of online survey research, online questionnaire authoring software packages, and web survey services. J. Comput.-Mediat. Commun. 10:JCMC1034. doi: 10.1111/j.1083-6101.2005.tb00259.x

[ref106] Yee N. (2006). The demographics, motivations, and derived experiences of users of massively multi-user online graphical environments. Presence Teleoperators Virtual Environ. 15, 309–329. doi: 10.1162/pres.15.3.309

[ref107] Yu E. Jung C. Kim H. Jung J. (2018). Impact of viewer engagement on gift-giving in live video streaming. Telemat. Inform. 35, 1450–1460. doi: 10.1016/j.tele.2018.03.014

[ref109] Zhang X. Xiang Y. Hao L. (2019). Virtual gifting on China's live streaming platforms: hijacking the digital gift economy. Chin. J. Commun. 12, 340–355. doi: 10.1080/17544750.2019.1583260

[ref110] Zhao Q. Chen C. D. Cheng H. W. Wang J. L. (2018). Determinants of live streamers' continuance broadcasting intentions on twitch: a self-determination theory perspective. Telemat. Inform. 35, 406–420. doi: 10.1016/j.tele.2017.12.018

[ref111] Zhou T. (2011). Understanding online community user participation: a social influence perspective. Internet Res. 21, 67–81. doi: 10.1108/10662241111104884

