# Supplementary material for: Cross-dimensional cultural identity: a multi-dimensional empirical investigation of VTuber audience behavior through the lens of theory of planned behavior
Source: Front Psychol. 2026 May 13;17:1675447. doi: 10.3389/fpsyg.2026.1675447 (PMC13213849; doi:10.3389/fpsyg.2026.1675447)
Supplement: Supplementary file 1 [file Supplementary_file_1.docx]

**APPENDIX**

**QUESTIONNAIRE**

**Perceived Entertainment**

(Vorderer et al.,2004; Hu et al.,2017):

**·**Watching the performances of this VTuber brings me a sense of joy and satisfaction.

**·**Engaging with this VTuber's content provides me with a rich and fulfilling entertainment experience.

**·**The live streams and video content produced by this VTuber are highly entertaining and captivating.

**·**Viewing this VTuber's content helps me relax and alleviates stress effectively.

**Virtual Identity Identification**

(Lu et al., 2021; Jin, 2012; Ratan and Dawson,2016):

**·**I find this VTuber's virtual avatar design to be compelling and appealing.

**·**I experience a strong sense of personal identification with this VTuber's virtual avatar.

**·**I believe this VTuber's virtual avatar demonstrates distinctive personality traits and charisma.

**·**I feel profound emotional resonance with this VTuber's virtual avatar.

**Interaction Quality**

(Wohn et al., 2018; Zhao et al., 2018; Hu et al.,2016):

**·**This VTuber provides prompt responses to audience comments and Danmaku (live chat messages), delivering timely feedback.

**·**This VTuber maintains a consistently high frequency of interaction with the audience.

**·**This VTuber creates a relaxed and positive atmosphere through audience engagement.

**·**This VTuber demonstrates focused commitment to audience interaction during live streams.

**Content Professionalism**

(Ohanian, 1990; Belanche et al., 2020; Vorderer et al.,2004):

**·**This VTuber demonstrates extensive professional knowledge and high-level competencies in their domain.

**·**This VTuber's livestream/video content exhibits thorough preparation and logical coherence.

**·**This VTuber conveys content with clarity and professional delivery methods.

**·**This VTuber produces well-crafted content with high overall presentation quality.

**Peer Influence**

(Sjöblom et al., 2017; Hilvert-Bruce et al., 2018; Hu et al.,2017):

**·**My peer group demonstrates substantial engagement with and interest in VTuber-related content.

**·**My peers perceive following VTubers as a trendy and worthwhile participatory activity.

**·**When peers engage in enthusiastic discussions about VTuber-related topics, I experience a perceived need for involvement.

**·**Peer recommendations significantly influence my selection and subscription decisions regarding new VTubers.

**Sense of Virtual Community, SOVC**

(Hilvert-Bruce et al., 2018; Hamilton et al., 2014):

**·**I frequently engage in interactive communications with other viewers in VTuber livestream channels, experiencing a collective atmosphere.

**·**I actively participate in collective fan activities within the VTuber community (e.g., coordinated cheering, fan support initiatives).

**·**I demonstrate comprehension and proficiency in utilizing the specific terminology and expression patterns characteristic of the VTuber fan community.

**·**Within the VTuber fan community, I exhibit proactive behavior in providing assistance and support to fellow community members.

**ACG Culture Identity**

(Tajfel, 1982; Ashforth and Mael, 1989):

**·**I demonstrate proficiency in the distinctive communicative patterns characteristic of ACG (Anime, Comic, and Games) culture and incorporate these expressions into daily interactions.

**·**I actively engage in ACG cultural community events, including anime conventions, online discussions, and fan support initiatives.

**·**I identify with and endorse the distinctive value systems and aesthetic principles manifested within ACG culture.

**·**I contribute to the dissemination of ACG cultural content through various forms of participation, including content creation, sharing, and commentary.

**Cross-Cultural Influence**

(Berry, 1997; Markus and Kitayama, 1991):

**·**By watching VTubers, I have gained a deeper understanding of cultural customs from different countries.

**·**Content from VTubers of various nationalities has effectively broadened my cultural perspective and enhanced my multicultural cognition.

**·**I can more accurately comprehend the cultural connotations and expressions demonstrated by VTubers from different countries.

**·**Through exposure to VTubers, I have gradually learned to understand and analyze phenomena from a multicultural perspective.

**Viewing Convenience**

(Davis, 1989; Cyr et al., 2006; Venkatesh et al., 2012):

**·**I can conveniently access VTuber content through multiple devices (such as smartphones, tablets, or computers).

**·**My physical environment is conducive to comfortably viewing VTuber live streams or recorded content.

**·**I have stable internet connectivity that enables smooth streaming of VTuber content.

**·**Bilibili's video-on-demand functionality allows me to flexibly watch VTuber content according to my schedule.

**Economic Capability**

(Sjöblom and Hamari, 2017; Zhao et al., 2018; Yu et al., 2018):

**·**I can consistently allocate budget for regular support of VTuber-related activities and content.

**·**I can comfortably afford the basic expenses required for accessing VTuber content (such as internet service fees).

**·**I possess sufficient financial resources to engage in regular monetary support behaviors (such as virtual gifting or membership subscriptions).

**·**I have additional financial capacity to purchase high-value VTuber merchandise or limited-edition products.

**Time Availability**

(Hu et al., 2017; Hilvert-Bruce et al., 2018):

**·**I can consistently allocate fixed time periods daily for consuming VTuber content.

**·**I have the flexibility to adjust my schedule as needed to watch VTuber's live streams or archived content.

**·**I can independently plan and manage my time to follow VTuber activities and updates.

**·**My schedule allows for sustained, long-term time investment in consuming VTuber content.

**Platform Accessibility**

(Lin and Lu, 2011; Gefen and Straub, 2000):

**·**Bilibili's search and recommendation algorithms effectively facilitate the rapid discovery of VTuber content aligned with user interests.

**·**The platform demonstrates robust technical performance and smooth loading capabilities when streaming VTuber content.

**·**The VTuber viewing interface on Bilibili exhibits intuitive design and operational efficiency.

**·**Bilibili provides comprehensive interactive functionalities for VTuber-related content, including comments, Danmaku (bullet screen comments), and monetary support features.

**Audience Attitude**

(Wongkitrungrueng and Assarut, 2018; Hilvert-Bruce et al., 2018):

**·**I consider VTuber content consumption to be an optimal form of entertainment.

**·**I perceive that engaging with VTuber content, including live streams and videos, provides beneficial value.

**·**I prioritize VTuber content consumption over alternative forms of entertainment.

**·**I perceive that the benefits derived from VTuber content consumption exceed the investments (time, effort, resources) made.

**Subjective Norm**

(Taylor and Todd, 1995; Dholakia et al., 2004):

**·**I take into consideration others' perspectives regarding my VTuber content consumption.

**·**I am inclined to modulate my VTuber viewing frequency when encountering disapproval from my immediate social circles.

**·**My family members' attitudes toward my VTuber viewing behavior influence my consumption patterns.

**·**I adjust my viewing habits in accordance with the prevalent societal perceptions of VTuber content.

**Perceived Behavioral Control**

(Ajzen, 2002; Ajzen, 1991):

**·**I possess effective control over the duration of my VTuber viewing sessions.

**·**I demonstrate comprehensive understanding of utilizing Bilibili platform for VTuber content consumption.

**·**I maintain the capability to resolve technical challenges that may arise during VTuber content viewing.

**·**I exhibit flexibility in adjusting my VTuber viewing schedule according to situational demands.

**Watch Intention**

(Hu et al., 2017; Hilvert-Bruce et al., 2018; Chen and Lin, 2018):

**·**I intend to maintain my engagement with VTuber live streams and archived content on Bilibili platform.

**·**I am willing to dedicate additional time to consuming VTuber content on Bilibili platform.

**·**I plan to maintain long-term attention to this VTuber's content updates.

**·**I express willingness to recommend my followed VTuber content to others.
